# Development and Application of Ruthenium(II) and Iridium(III) Based Complexes for Anion Sensing

**DOI:** 10.3390/molecules28031231

**Published:** 2023-01-27

**Authors:** Ambreen Rashid, Sahidul Mondal, Pradyut Ghosh

**Affiliations:** School of Chemical Sciences, Indian Association for the Cultivation of Science, 2A & 2B Raja S. C. Mullick Road, Kolkata 700032, India

**Keywords:** anion sensing, luminescent chemosensors, ruthenium(II)/iridium(III) complexes

## Abstract

Improvements in the design of receptors for the detection and quantification of anions are desirable and ongoing in the field of anion chemistry, and remarkable progress has been made in this direction. In this regard, the development of luminescent chemosensors for sensing anions is an imperative and demanding sub-area in supramolecular chemistry. This decade, in particular, witnessed advancements in chemosensors based on ruthenium and iridium complexes for anion sensing by virtue of their modular synthesis and rich chemical and photophysical properties, such as visible excitation wavelength, high quantum efficiency, high luminescence intensity, long lifetimes of phosphorescence, and large Stokes shifts, etc. Thus, this review aims to summarize the recent advances in the development of ruthenium(II) and iridium(III)-based complexes for their application as luminescent chemosensors for anion sensing. In addition, the focus was devoted to designing aspects of polypyridyl complexes of these two transition metals with different recognition motifs, which upon interacting with different inorganic anions, produces desirable quantifiable outputs.

## 1. Introduction

Anions play vital roles in numerous physiological and industrial processes [1,2,3,4,5,6,7]. In addition, anionic species are omnipresent in the environment and act as an essential component to sustain growth and/or as pollutants [8,9,10]. So, the selective detection and sensing of anions are of immense interest in supramolecular chemistry. The more complex geometries, larger size, pH dependency, and higher hydration energies of the anions compared to cations make selective anion binding an additional challenging task. In the last few decades, significant progress has been made in the area of designing receptor molecules for the detection and quantification of anions [11,12,13,14,15,16,17].

**Figure 1:**
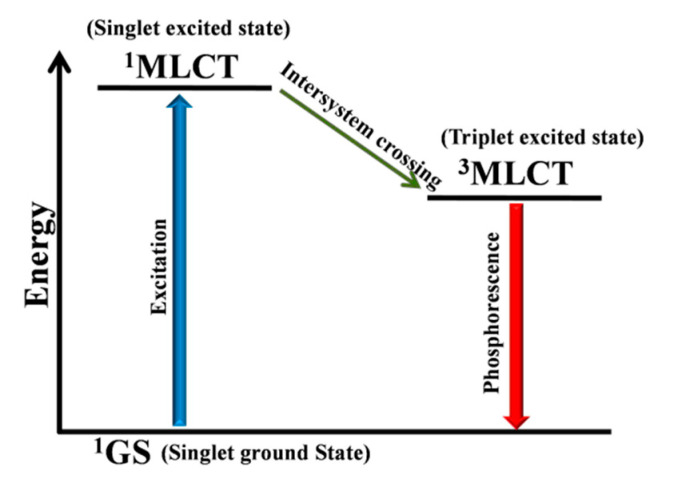
Simplified Jablonski diagram of Ru^II^/Ir^III^ complexes showing triplet-state emission mechanism.

In recent years, the development of phosphorescent compounds with unique photophysical properties is growing as a wide area of research. Such organometallic complexes usually consist of transition metal ions of the second and third row, such as rhenium(I), ruthenium(II), iridium(III), platinum(II), osmium(II) and gold(I), and organic ligand frameworks [18,19,20,21,22,23]. These metal complexes usually have a range of excited states, such as ligand-to-ligand charge transfer (LLCT), metal–metal-to-ligand charge transfer (MMLCT), ligand-to-metal charge transfer (LMCT), intra-ligand charge transfer (ILCT), metal-to-ligand charge transfer (MLCT), and ligand-to-metal–metal charge transfer (LMMCT) [19,24,25,26]. The emission properties of these complexes can be varied by coordinating different ligands and varying the local environments. Upon photoexcitation, the strong spin-orbit coupling in heavy metals facilitates rapid inter-conversion of the singlet excited states to the triplet excited state via effective intersystem crossing states. This triplet-state emission is responsible for the intense and long-lived phosphorescence in transition metal complexes [24] (Figure 1).

Among the various transition metal compounds, Ru(II) and Ir(III) complexes are receiving significant interest because of their application as molecular receptors and probes for anion sensing. In this regard, the fundamental criteria for the effective synthesis of such complexes are (i) high quantum yields at room temperature, (ii) appropriately large Strokes shift which limits inner filter effects, (iii) decent photochemical and photo-physical stability, (iv) easily tunable-photophysical properties with readily accessible near-infrared (NIR) emission,(v) ease of synthesis through ligand modification, and (vi) longer lifetime of emission which limits interference from autofluorescence [27,28].

A widely followed approach for the design of receptor host molecules is that the host must provide a binding site where the anion can coordinate/bind with the receptor molecule. Various supramolecular interactions, such as electrostatic interactions [29,30,31,32,33], hydrogen bonding [34,35], halogen bonding [36,37,38,39], anion -π interactions or π-π interactions [40,41,42,43,44], or the formation of a new host-guest conjugate species [13] (chemodosimeter) are commonly operational between the receptor molecule and the anion. Moreover, the anion binding event must be quantified by a detectable signaling output response which can be followed optically (naked eye, fluorescence, or phosphoresce color change), spectroscopically (through NMR, UV-Vis, PL), electrochemically (through an ion-selective electrode or cyclic voltammetry (CV)), through a sol-gel transformation or isothermal titration calorimetrically. The incorporation of an optical or electronic transducer into the receptor molecule responsive to a particular anion serves the above process and assists not only in detection but also in sensing.

Some of the review articles about transition metal-based chemosensors for anions, cations, and small molecules have been published [25,26,45,46,47,48,49]. However, some of them only focused on a specific motif-based (polar -NH moiety-based [46]; 1,10-phenanthroline-based [47]), while some of them published specific anions binding ruthenium or iridium complexes [48,49]. Here, this review seeks to summarize the recent advances in the development of luminescent ruthenium (II) and iridium (III) polypyridyl complexes for the purpose of anion sensing with different anion recognition motifs, which produces desirable results upon interacting with different inorganic anions. This review will focus on some recent development of polypyridyl complexes of ruthenium (II) and iridium (III) and their application as anion sensors.

## 2. Discussion

### 2.1. Ruthenium(II) Based Complexes for Sensing of Anion

These Ru(II) polypyridyl complexes reviewed as better chromophores have attracted tremendous interest owing to their notable large Stokes shift, excited state lifetime, and photophysical, electrochemical, and redox properties [46,50]. The amalgamation of ruthenium with a bipyridine (bpy) or phenanthroline (phen) moiety and a felicitous ligand could provide a binding site and a signaling unit for anions. In 1993, Beer and co-workers first reported [Ru(bpy)_3_]^2+^ complexes for anion sensing where amide substituents in bipyridine moiety acted as the anionic recognition site [51].

#### 2.1.1. Triazole/Iodo-Triazole-Based Ru(II) Complexes as Chemosensors

In this part, we have concisely described triazole/iodo-triazole-based ruthenium(II) complexes for the “turn on” detection of anions through solitary C-H/C-I^…^ anion interaction. Triazole C-H is acidic in nature, and its incorporation with ruthenium metal makes it a suitable recognition site for anions. The previously described Ru(II) polypyridyl complexes worked as wonderful luminescent chemical sensors because of their distinctive optical properties such as large Stokes shift, extended excited-state lifetime, and excitation at visible region. 

**Figure 2:**
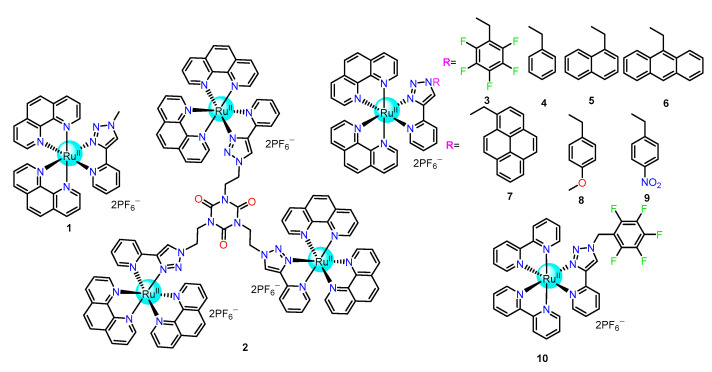
Structures of triazole-based Ru(II) complexes **1**–**10**.

Since 2014, our group has systematically developed bis-heteroleptic ruthenium(II) complexes for selective sensing, recognition, and extraction of H_2_PO_4_^−^ and HP_2_O_7_^3−^ anions. In this regard, mononuclear Ru(II) complex **1** and trinuclear electron-deficient cyanuric acid template-based Ru(II) complex **2** containing pyridine triazolium and phenanthroline units were synthesized for selective sensing of H_2_PO_4_^−^ and HP_2_O_7_^3−^ (Figure 2) [52,53]. Both of these complexes showed distinctive MLCT charge transfer absorption bands that popped up at 403 nm [Ru(dπ)→phenanthroline] and 445 nm [Ru(dπ)→ triazole pyridine]. A weak luminescence band was observed at 590 nm upon excitation at either 403 or 445 nm for both complexes. Qualitative analysis of these two receptors by ^1^H NMR and UV-PL spectroscopies resulted in selective recognition of phosphates amid other examined anions (e.g., I^−^, Br^−^, Cl^−^, F^−^, NO_3_^−^, HSO_4_^−^, CH_3_CO_2_^−^, ClO_4_^−^, PhCO_2_^−^, HCO_3_^−^, etc.). Further PL titration of **1** showed an increase in emission intensity by 6-fold for H_2_PO_4_^−^ and 3-fold with HP_2_O_7_^3−^ as compared to the free receptor, whereas **2** depicted 20-fold and 7-fold enhancement upon HP_2_O_7_^3−^ and H_2_PO_4_^−^ binding, respectively. Through the –C-H unit of the triazole moiety mononuclear Ru(II) complex, **1**, selectively binds with the singly charged and comparatively smaller H_2_PO_4_^−^ anion, as established from X-ray crystallographic analysis. On the contrary, the trinuclear Ru(II) complex **2** was more flexible compared to **1**, had numerous anion recognition sites and therefore preferred to bind with the larger and more negatively charged HP_2_O_7_^3−^ anion. Further, **2** exhibited more selectivity towards higher phosphate analogs (such as ATP, ADP, and AMP) over HP_2_O_7_^3−^ anions when a 10 mM concentrated solution of 10% Tris-HCl buffer in acetonitrile (having 1/9, *v*/*v*) medium was used. Excitingly, Job’s plot analysis revealed **1** and **2** formed 1:1 and 1:3 (H:G) complexes, respectively, with phosphates. The overall binding constant values in the presence of H_2_PO_4_^−^ for **1** and **2** were calculated to be 5.28 × 10^4^ M^−1^ and 6.76 × 10^13^ M^−3^, respectively, and these values also revealed the trend of reversal of phosphate selectivity by **1** and **2**. Remarkably, the lifetime of **2** was raised by 28-fold and 8-fold in the presence of HP_2_O_7_^3−^ and H_2_PO_4_^−^, respectively, and it could be used as a potential sensor owing to their higher excited state lifetime.

In addition, our group developed an array of bis-heteroleptic Ru(II) complexes **3**–**10** (Figure 2), having the pyridine triazole unit as the anion binding site along with various other substituents which were involved in a meticulous comparative investigation for the development of superior selective probes for phosphates [54]. All these complexes showed photo-physical properties, which were similar to **1** and **2**. UV/Vis spectra of these complexes (**3**–**9**) showed similar intense broad absorption peaks in the region 400–405 nm and 440–445 nm attributed to MLCT charge transfer from Ru-to-phenanthroline and Ru-to- triazole pyridine, respectively. In the case of **10**, the absorption band appeared at 412 nm (Ru-to-bipyridine transition) and 440 nm (Ru-to- triazole pyridine transition). Probe **6** showed characteristic peaks at λ_max_391, 370, and 351 nm for the anthracene unit, whereas seven peaks at λ_max_ 343, 327, and 313 nm were attributed to the pyrene unit, owing to π-π* transitions in both cases. Qualitative analysis showed that all these receptors were selective towards phosphates only, even in the presence of other competitive anions. Further, the gradual addition of phosphates to probes **3**–**9** individually resulted in a significant perturbation of the emission spectra reflected by increased emission intensity. In addition, the emission band was observed to be red-shifted by 15 nm and 50 nm in cases **6** and **7**, respectively. In complexes **3**–**10**, the acidic nature of the triazole C-H bond was changed by alternating the pendent substituents from electron-withdrawing to electron-donating, followed by polyaromatic substituents. Receptors **4**–**9** showed 5 to 10-fold enhancements of emission intensity in the presence of H_2_PO_4_^−^ whereas **3** and **10** displayed ~22-fold and ~19-fold increases, respectively. Thus, it can be concluded that the pentafluorophenyl moiety in the substituent makes **3** and **10** comparatively superior sensors for phosphates accredited to the rigidification of molecular backbone assisted by supramolecular self-assembly, hydrogen bonding and anion-π interactions. The Job’s plot analysis suggested 1:1 (H:G) binding of receptors with H_2_PO_4_^−^ where binding constant values ranged from 10^4^ to 10^6^ M^−1^. Among the receptors, receptor **3,** having a pendent pentafluorophenyl moiety, showed the highest binding constant of 2.64 × 10^6^ M^−1^ and a very low detection limit of ~0.013 μM for H_2_PO_4_^−^ Furthermore, the ^1^H NMR experiment revealed that the sharp singlet corresponding to the triazole C-H was found to be shifted to a more downfield region in the presence of H_2_PO_4_^−^. Again, ^19^F NMR experimental results of **3** and **10** exposed an additional anion-π interaction between pentafluorophenyl moiety and H_2_PO_4_^−^ which enabled them to be used as efficient sensors for phosphates. In addition, macroscopic morphological studies such as Transmission electron microscopy (TEM), Diffusion-ordered spectroscopy (DOSY) NMR experiments, and Dynamic light scattering (DLS) results disclosed that supramolecular aggregation occurred in solution and the solid state for all these complexes with H_2_PO_4_^−^. The phosphate adducts of **3** and **10**, as compared to other complexes, showed a maximum decrease in diffusion coefficient, i.e., from 7.188 × 10^−10^ to 1.659 × 10^−10^ m^2^/S for **3** and from 1.136 × 10^−9^ to 2.179 × 10^−10^ m^2^/S for **10**. Thus, these macroscopic data results confirm the formation of higher-order supramolecular aggregates in solution, and this aggregate formation is assisted by anion-π interaction. Finally, the single crystal X-ray structure of phosphate adduct of **3** established the formation of supramolecular aggregates via anion-π and strong directional hydrogen-bonding interaction between triazole C-H and oxygen atoms of H_2_PO_4_^−^. Electron-deficient pentafluorophenyl substituent, **3** was reported as a superior probe for H_2_PO_4_^−^ in terms of a higher binding constant, larger enhancement of emission intensity, and lower detection limit over another aryl- and polyaromatic-substituted analogs (**4**–**10**). The superiority of **3** was realized because of the pentafluorophenyl substituent, which helped in the formation of supramolecular aggregation via both anion-fluorine/anion-π non-covalent and hydrogen bonding interactions. 

**Figure 3:**
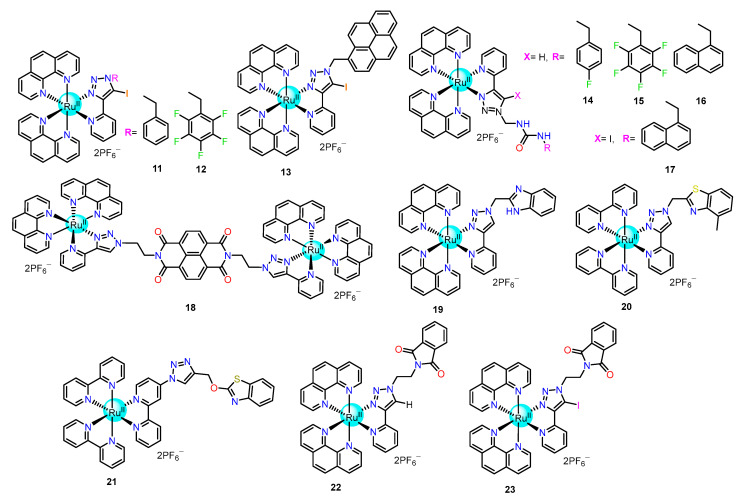
Structures of triazole-based Ru(II) complexes **11**–**23**.

The significant phosphate selectivity via the C–H⋯ anion interactions by Ru (II)-polypyridyl complexes encouraged us to design halogen bonding-based receptors **11** and **12** (Figure 3) by replacing the hydrogen atom of C-H triazole bond with iodine [55,56]. Complexes **11** and **12** containing the iodotriazole (halogen bond unit) with phenyl and pentafluorophenyl (π-acidic) units as the pendent moiety for **11** and **12**, respectively, showed superior dihydrogen phosphate sensing over their hydrogen bonding analogs. These receptors resembled the hydrogen bonding analogs in terms of UV-Vis absorption, emission spectra, and binding studies. The acetonitrile solution of receptors **11** and **12** showed ~16-fold and ~25-fold increments of emission intensities, respectively, in the presence of dihydrogen phosphate. The calculated binding constants for **11** and **12** were observed as 1.95 × 10^5^ and 2.76 × 10^6^ M^−1^, respectively, and the limit of detection of H_2_PO_4_^−^ were 0.018 µM and 0.011 µM respectively. Furthermore, the DOSY NMR experiment and crystal structure analysis of receptors **11** and **12** exposed the formation of supramolecular polymeric structure in both solution and solid states through halogen bonding interaction and made them more sensitive and selective sensors for phosphates.

**Figure 4:**
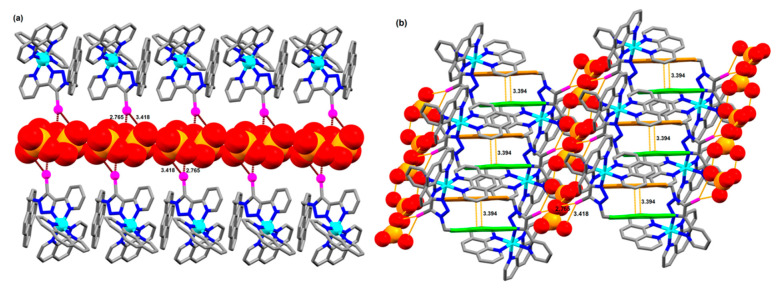
X-ray crystal structure of H_2_PO_4_^−^ adduct of **13** where supramolecular polymeric chain propagated via (**a**) C-I···O interaction through halogen bond; (**b**) combined C-I···O and π-π stacking interactions. [Adapted from reference [57]; Copyright © 2023, American Chemical Society].

Between these two, **12** was found to be a superior sensor over **11** due to the presence of the pentafluorophenyl pendent moiety, which forms anion-π interaction with the dihydrogen phosphates, which additionally assisted in the formation of supramolecular aggregation. The DLS experiment further confirmed the formation of supramolecular aggregates in solution as the hydrodynamic diameter of free receptor **12** increased from ~130 nm to ~869 nm upon the addition of phosphates. In addition, due to the strong and highly directional nature of halogen bonding interaction, **12** could also be used as a perrhenate receptor in acetonitrile. To establish the origin of selective phosphate sensing phenomena by Ru(II)-polypyridyl, halogen bonding interaction-based receptor **13** (Figure 3) was designed, which contained an iodo-triazole unit as the anion binding unit and pendent pyrene as the fluorophoric as well as π-π stacking unit [57]. This receptor also showed a similar absorption and emission at 405 nm and 590 nm, respectively, and similar anion binding properties as observed previously. The presence of the pyrene unit caused characteristic absorption peaks at 314, 327, and 343 nm to appear, and the excitation at any of these peaks resulted in emission at 377, 390, 417, and 470 nm. These emission peaks corresponding to the pyrene moiety increased by ~2.8 fold upon the addition of H_2_PO_4_^−^, and so this receptor could be used as a dual sensor for dihydrogen phosphates. Job’s plot analysis showed a 1:1 (H:G) interaction, and the calculated binding constant and detection limits were 8.1 × 10^4^ M^−1^ and ~0.10 μM, respectively. The solution state investigation was performed by DOSY NMR and DLS experiments. The results showed almost a 34% reduction in the diffusion coefficient and an increase in hydrodynamic radius from 140 nm to 889 nm of free probe **13** upon the addition of 1 equiv. of H_2_PO_4_^−^ which additionally affirmed the formation of supramolecular aggregates into the solution. The macroscopic investigations by Scanning electron microscope (SEM) studies imparted that the free receptor formed a spherical vesicular structure with an approximate size of 200 nm, which transformed into a rod-like structure with an approximate size of 30 μM. Finally, solid-state PL and crystal structure analysis confirmed the formation of solid-state supramolecular polymeric assembly directed by strong C–I··· O halogen bonding interaction between the iodine of iodo-triazole unit and the oxygen atom of phosphate, π-π stacking of pyrene unit, and polymeric phosphates chain (Figure 4). The formation of supramolecular aggregates might rigidify the molecular backbone and cease the non-radiative decay, which subsequently increases the emission intensity.

After the efficacious establishment of the halogen bond into the triazole moiety of Ru(II)-polypyridyl, a pendent urea moiety was amalgamated with the triazole unit to enhance the binding ability and extraction applicability of phosphates. For this purpose, **14**–**17** (Figure 3) were synthesized for recognition and sensing of phosphate via combined –C-H/–NH or –C-I/–NH anion hydrogen bonding or halogen bonding interactions [58,59,60]. A detailed comparative study exposed that the naphthyl urea-modified analogs **16** and **17** had the optimal acidity of the urea –NH protons which made them better sensors for H_2_PO_4_^−^ compared to other urea and non-urea hydrogen analogs. Again, **17** exhibited the highest binding affinity, sensitivity, and lowest detection limit in comparison to **16** due to the combined effect of halogen bonding of idodo-triazole (C-I) moiety and hydrogen bonding interaction of urea –NH protons. The binding order of the receptors followed the order **17** > **16** > **14** > **15**. The presence of acidic urea moiety enabled all these receptors to bind with other oxyanions, such as carboxylates (e.g., CH_3_CO_2_^−^ and C_6_H_5_CO_2_^−^).

Recently our group developed a molecular donor-acceptor-donor triad **18** (Figure 3), which encompassed an electron-scarce 1,4,5,8-naphthalene tetracarboxylic diimide (NDI) moiety covalently linked to two triazole-pyridine-based bis-heteroleptic Ru(II) complex of 1,10-phenanthroline [61]. The electron-poor NDI unit acted as an anion-binding host through anion-π interaction. Meticulous studies in solutions such as NMR, UV-Vis, and EPR displayed that F^−^ and CN^−^ selectively interacted with the NDI unit to form a radical anion, NDI^•−^ which was further reduced to dianionic NDI^2−^ only in the presence of F^−^. Remarkably, this triad exhibited “OFF-ON” phosphorescence properties in the presence of fluoride ions by re-electing the ^3^MLCT emission of Ru(dπ)→triazole pyridine/phenanthroline whereas cyanide failed to show such similar results.

Similarly, Khatua and his group also developed benzimidazole substituted 1,2,3-triazole pyridyl Ru(II) complexes **19** and **20** (Figure 3), which imparted them as hydrogen bonding-based phosphates sensors [62,63]. Triazole–CH proton displayed downfield shifts in the ^1^H NMR experiment that indicated its interaction with phosphate ions. Receptor **19** showed around a 10-fold enhancement of emission intensity (at 583 nm), whereas receptor **20** showed a 57-fold increment of the same (at 575 nm) upon subsequent addition of two equiv. of H_2_PO_4_^−^. The reported association constants for **19** were noted as 6.8 × 10^3^ M^−1^ and 3.3 × 10^3^ M^−1^, whereas the limit of detections (LOD) were 5.19 and 5.73 ppb for HP_2_O_7_^3−^ and H_2_PO_4_^−^, respectively. Similarly, **20** exhibited strong binding towards HP_2_O_7_^3−^/H_2_PO_4_^−^ and was found to be superior to **19** due to higher binding constant (Ka = 8.7× 10^5^ M^−1^ and 2.4 ×10^5^ M^−1^ for H_2_PO_4_^−^ and HP_2_O_7_^3−^ respectively) and lower LOD (0.48 μM and 0.43 μM for H_2_PO_4_^−^ and HP_2_O_7_^3−^ respectively).

Again, Anandan et al. reported a polypyridine complex of Ru(II), **21** (Figure 3) comprising of 1,2,3-triazole linker holding a benzothiazole unit for the detection of phosphates and Cu^2+^ ions [64]. The ^1^H NMR titration exhibited that basic anions such as HP_2_O_7_^3−^ and H_2_PO_4_^−^ upon interacting with the acidic triazole C–H showed significant sharp downfield shift (Δδ = 0.15 ppm) and broad downfield shift (Δδ = 0.15 ppm) of triazole C-H proton upon addition of 1.0 equiv. of H_2_PO_4_^−^, and HP_2_O_7_^3−^, respectively. Further, the PL titration of **21** exhibited a 2.3 and 1.9-fold increase in emission intensity in the presence of HP_2_O_7_^3−^ and H_2_PO_4_^−^, respectively, in acetonitrile. The association constants were obtained from PL titration, providing values of 7.7 × 10^5^ M^−1^ and 4.2 × 10^5^ M^−1^, whereas detection limits were 0.22 μM and 0.31 μM for H_2_PO_4_^−^ and HP_2_O_7_^3−^, respectively. Again, receptor **21** showed one reversible oxidation peak at 1.13 V in Ag/AgCl reference electrode, which corresponds to Ru^II^/Ru^III^ oxidation. In addition, a new oxidation peak at 0.90 V, accompanied by an anodic shift of the oxidation peak at 1.13 V, was found due to the addition of 1 equiv. of H_2_PO_4_^−^ and HP_2_O_7_^3−^.

This group further developed non-luminescent hydrogen and halogen-bonded metalloreceptors **22** and **23** (Figure 3), respectively, where selective turn-on luminescence sensing was observed upon the addition of phosphate anions in acetonitrile [65]. Both metalloreceptors have brilliant sensing properties for H_2_PO_4_^−^ and HP_2_O_7_^3−^ over other common inorganic anions in acetonitrile. The initial lifetimes of the non-luminescent receptors **22** and **23** were increased from ~2 ns to ~54 ns and from ~2 ns to ~61 ns, respectively, for the H_2_PO_4_^−^ anion. Comparative studies (such as - ^1^H NMR titration, ^31^P NMR titration, lifetime studies, and electrochemical experiments) revealed that non-covalent interactions (halogen/hydrogen bonding) played a vital role in the sensing of phosphates and, moreover, the halogen bonding exhibited better recognition properties over hydrogen bonding. The LOD value of H_2_PO_4_^−^ for **22** and **23** was calculated at 79 nM and 48 nM, respectively.

#### 2.1.2. Imidazole/Benzimidazole-Based Ru(II) Complexes as Chemosensors

Since 2005, imidazole/benzimidazole-based ruthenium(II) complexes, especially bi(benz)imidazole ligand-based, were potentially used as anion receptors as their chromogenic and redox properties enabled them to be appropriate candidates for optical molecular recognition [66,67]. Imidazole and its derivatives have been broadly exploited for the recognition and sensing of basic anions such as F^−^, H_2_PO_4_^−^, CH_3_COO^−^, etc. In general, the C-H or N-H moiety in the imidazole unit acted as recognition sites. Due to the more acidic nature of polarized -NH, it served as a better recognition center compared to the C-H group of imidazole. Further, the acidity of the -NH group could be easily altered by varying the electronic properties of neighboring substituents. In general, the recognition process proceeds via hydrogen bonding interaction between the acidic -NH proton of imidazole and basic anions or via the deprotonation of the imidazole -NH by the anions [47].

**Figure 5:**
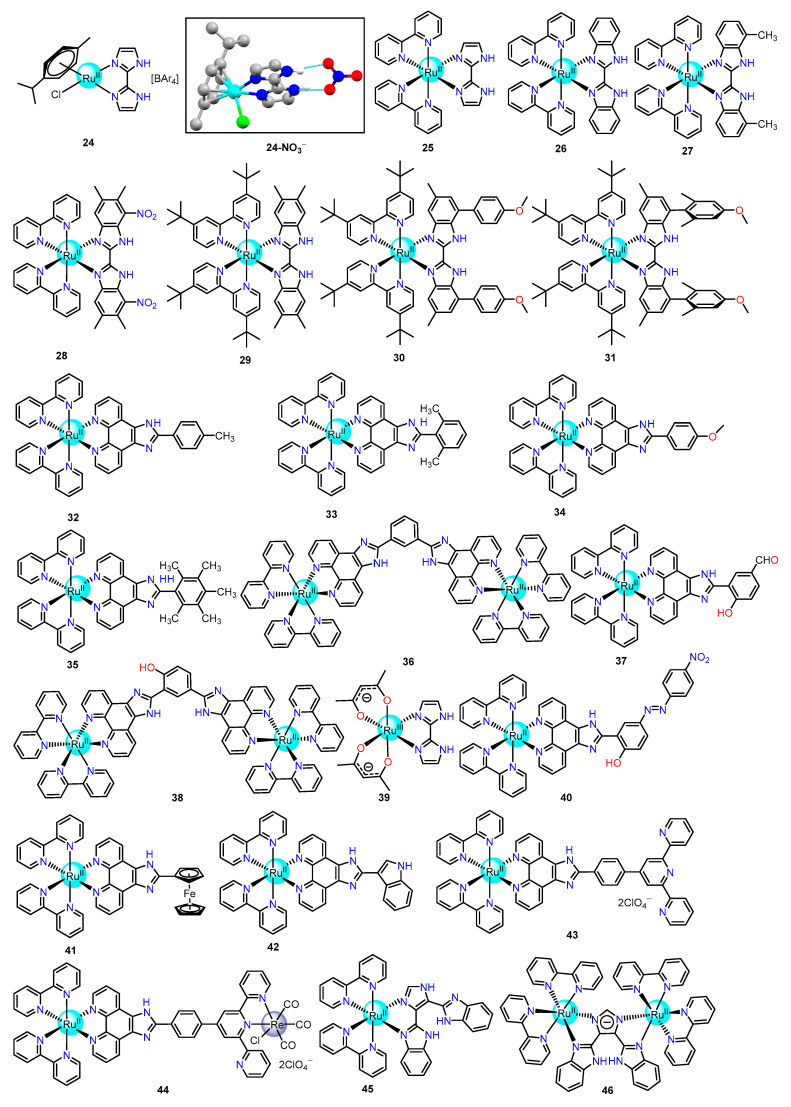
Structures of biimidazole/imidazole-based Ru(II) complexes **24**–**46** and the crystal structure of **24**-NO_3_**^−^** complex. [Adapted from ccdc no.279202].

McPartlin and co-workers first investigated the solution state properties of chlorido para-cymol ruthenium (II) biimidazole complex **24** (Figure 5) and established strong, non-selective interactions with some common inorganic anions such as- Cl^−^, Br^−^, NO_3_^−^, I^−^ or ReO_4_^−^ [68]. The anion binding was investigated by ^1^H NMR titration with the above-mentioned inorganic anions, and the shift of the –NH proton was studied. According to the basicity of the anions, it was expected that Cl^−^ and Br^−^ would bind strongly with the receptor compared to NO_3_^−^ and HSO_4_^−^. However, the NO_3_^−^ ion showed higher binding affinity due to the structural complementarity confirmed by the single-crystal X-ray structure (Figure 5).

The Ye group reported a series of bis-heteroleptic Ru(II)complexes, **25**–**28** (Figure 5) containing 2,2-bisimidazole as the recognition site for anion and Ru(II)-bipyridine moiety as the signaling unit [69,70,71,72]. Remarkably, the bisimidazole acted as a bifunctional unit due to the presence of two lone pairs of electrons for coordination to the central metal ion, and the two imidazole –NH groups provided exclusive hydrogen bond for second coordination sphere recognition of anions. The detailed analysis demonstrated that the photophysical and electrochemical properties of **25**–**28** depend on the protonated state of the –NH group of the bisimidazole moiety. The acidic nature of the bisimidazole –NH group may grant a hydrogen bonding interaction state, mono-deprotonated state, and double deprotonated state in the presence of anions, and these states were governed by numerous factors such as the acidity of the metallo-receptor, basicity of anions, strength, and the number of hydrogen bonds. Metallo-receptor **25** offered two –NH protons toward I^−^, Br^−^, Cl^−^, H_2_PO_4_^−^, CH_3_COO^−^, HSO_4_^−^, and NO_3_^−^ for hydrogen bonding interactions, whereas only one of the –NH proton is deprotonated upon excess addition of CH_3_COO^−^ which altered the color of the solution from yellow to orange-brown. Conversely, fluoride formed highly stable HF_2_^−^ by deprotonating both the acidic protons of bisimidazole –NH and altered the color of the solution from yellow to orange-brown followed by violet.

Again, ruthenium(II) 2,2- bisbenzimidazole complex **26** showed a similar type of anion binding properties towards I^−^, Br^−^, Cl^−^, H_2_PO_4_^−^, CH_3_COO^−^, HSO_4_^−^, and NO_3_^−^ as observed in the case of **25**. The 2,2-bisbenzimidazole unit in **26** operated as electron-withdrawing groups and made it a more acidic receptor (pKa 5.7) as compared to **25** (pKa 7.2). In the presence of both F^−^ and CH_3_COO, **26** formed a mono-deprotonated complex followed by the double-deprotonated complex at low anion and high anion concentrations, respectively. On the contrary, distinct responses were observed in the case of substituted acetate anions such as CCl_3_COO^−^ and CF_3_COO^−^. Later this group introduced receptor **27**, containing 7,7-dimethyl-2,2-bisbenzimidazole ligand, to make an intermediate acidic complex (pKa 6.2) between **25** and **26**. This moderately acidic complex displayed significant sensing properties towards both the weakly basic anions (I^−^, Br^−^, Cl^−^, H_2_PO_4_^−^, HSO_4_^−^, and NO_3_^−^) as well as strongly basic anions (F^−^ andCH_3_COO^−^). The emission intensity (at 617 nm) was improved by about 35–40% upon binding weakly basic anions through hydrogen bonding interactions which rigidified the molecular backbone and ceased the non-radiative decay of the ^3^MLCT state. On the contrary, the addition of strongly basic anions (such as CH_3_COO^−^ and F^−^) caused the luminescence intensity of **27** to be quenched through the formation of doubly deprotonated species. Furthermore, the acidity of –NH protons was increased by incorporating the –NO_2_ group in the 2,2-bisbenzimidazole unit, which could form intramolecular hydrogen bonding and act as anion receptors. In this regard, the Ye group developed the ruthenium(II) bisimidazole-like complex **28,** which showed a strong binding affinity towards Br^−^and Cl^−^, a weaker affinity towards I^−^ and no affinity towards other basic anions such as HSO_4_^−^ and NO_3_^−^. Interestingly, deprotonation of the bisimidazole –NH of **28** was observed in the presence of strong basic anions, such as CH_3_COO^−^ and F^−^.

In addition, Rau et al. developed ruthenium(II) bibenzimidazole complexes **29**–**31** (Figure 5) for the recognition and sensing of halide anions [73]. All the complexes **29**–**31** showed a similar type of bathochromic shift of the ^3^MLCT band along with luminescence quenching upon the addition of F^−^ and OH^−^. Due to p-anisyl substituents at 4,4’-positions of **30** and **31**, a specified binding pocket for anion is generated at peripheral diamine functions. It should be noted that rotation of the aryl rings provides structural flexibility to **30,** which allowed the positively polarized C-H edges to bind with the anion actively. On the contrary, the rotation of the p-anisyl moieties in **31** is prevented due to the presence of four methyl substituents, and thus, it formed a rigid structural conformation. Due to this structural rigidity,**31** displayed an up-field shift of –NH proton, whereas a distinct shift of –NH signals (Δδ= 3–5 ppm) was observed in the case of other receptors in the presence of I^−^, Br^−^, and Cl^−^. 

**Figure 6:**
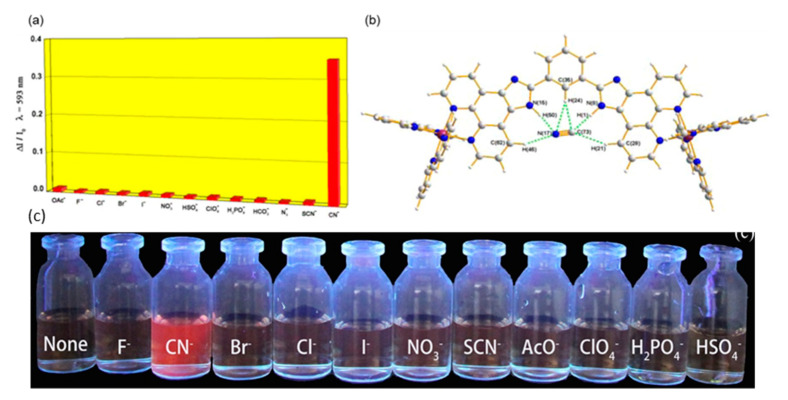
(**a**) Relative emission response of **36** (1.0 μM in pH = 7.00; 0.02 M HEPES buffer) at 593 nm in the presence of 5 mM potassium salts of the aforesaid anions; (**b**) Optimized structure of **36**–CN at the B3LYP/6−31G* level for H, C, and N atoms and the SDD for Ru. [Adapted from reference [74]; Copyright © 2023, American Chemical Society]. (**c**) Emission color change under 365 nm UV lamp of **37**–Cu^2+^ (10 μM) in the presence of 20 equiv. of different anions. [Adapted from reference [75]; © 2023 Elsevier B.V. All rights reserved].

Further, the Ye group assimilated the idea of multipoint hydrogen bonding interactions to improve the probability of anion binding affinity and solubility in water. In this regard, a set of complexes **32**–**36** (Figure 5) were prepared and used for selective recognition of CN^−^ in water [74]. Amongst all the anions, only CN^−^ perturbed the emission spectra of **32** and **36** via the formation of hydrogen bonding with the receptor at physiological conditions in the water. Bimetallic ruthenium(II) complex **36** showed higher affinity and sensitivity towards CN^−^ in water (Figure 6). It might be due to the appropriate pKa’s and C-shape cavity structure of complex **36,** which provided multiple hydrogen bonding interactions towards CN^−^. The theoretical investigation also suggested CN^−^ was capped inside the C-shape cavity of the receptor through five-point hydrogen bonding interaction that provides an energy-minimized structure (Figure 6). The calculated binding constant of **36** was 2.5 times higher, and the detection limit was 20 times lower than **32**. 

Again, hydroxyphenyl imidazo appended ruthenium (II) complexes **37** and **38** (Figure 5) were presented by Ma et al. for “turn-off-on” sensing of CN^−^ [75]. The luminescence properties of both complexes were instantaneously quenched upon binding with Cu^2+^ in an aqueous buffer solution (20 mM HEPES, pH = 7.2) containing 1% (*v*/*v*) acetonitrile. Non-luminescent **37**-Cu^2+^ and **38**-Cu^2+^ adducts were formed and could selectively and sensitively detect the very low concentration of CN^−^ in 99% aqueous solutions through a “turn-on” fluorescent response (Figure 6). The calculated LOD for CN^−^ was 0.36 μM for **37**-Cu^2+^ and 0.87 μM for **38**-Cu^2+^, which were lesser than the World Health Organization (WHO) recommendation value (maximum admissible concentration of 1.90 μM in drinking water). So, these ruthenium (II) complex-based ensembles could be used as potential and efficient cyanide ion sensors in nearly 100% aqueous solutions.

**Scheme 1:**
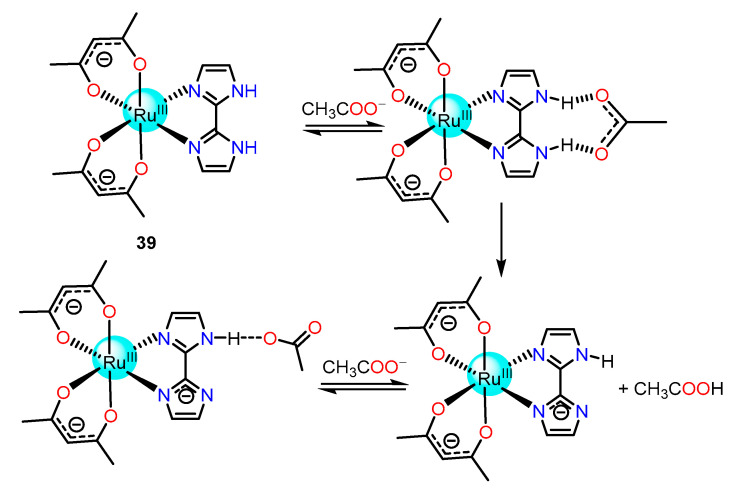
Mode of hydrogen bonding interaction between **39** and CH_3_COO^−^.

Colorimetric recognition of basic anions, for example, F^−^, H_2_PO_4_^−^, HSO_4_^−^, Br^−^, Cl^−^, I^−^, and CH_3_COO^−^ by non-luminescent paramagnetic bis(acetylacetonate) biimidazole Ru(III) complex **39** (Figure 5), was reported by Lahiri and his group [76]. A gradual color change of receptor **39** was observed in the presence of basic anions caused due to deprotonation, whereas weakly basic anions such as halides and sulphate formed only minor hydrogen bonding with the imidazole –NH proton of **39** (Scheme 1).

Again, azocoupled salicylaldehyde imidazole-based Ru(II) complex **40** (Figure 5) was documented by Khanmohammadi et al. for selective detection of basic anions such as F^−^, H_2_PO_4_^−^, CH_3_COO^−^, etc. in DMSO [77]. UV-Vis spectra of **40** showed a decrease in band intensity at 355 nm and simultaneously the debut of a new band at 550 nm, followed by an alteration of the colour of the solution from light orange to dark brown. In contrast, the emission intensity at 735 nm was enhanced upon the addition of anions to the complex.

Molina et al. reported ruthenium(II)–polypyridyl complex **41** (Figure 5), containing ferrocene-imidazophenanthroline ligand and ruthenium bipyridine moiety for extremely selective detection of Cl^−^ over all other anions [78]. The sensing studies of this heterobimetallic ruthenium (II) complex were carried out in two ways: (i) cathodic redox shift of the Fe(II)/Fe(III) couple (ΔE_1/2_ = −80 mV) without changing the oxidation wave of the ruthenium (II) center; and (ii) a noticeable enhancement of red emission (CHEF = 30). The ^1^H NMR,^31^P NMR, and all the photo-physical and electrochemical data strongly suggested imidazole –NH proton bind with Cl^−^ through hydrogen bonding interactions.

**Scheme 2:**
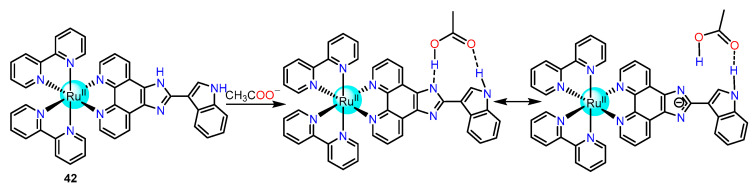
Mode of hydrogen bonding interaction between **42** and CH_3_COO^−^.

Wang and co-workers synthesized Ru(II) complex **42** (Figure 5), encompassing both imidazole and indole groups, which acted as selective and naked eye detectors of CH_3_COO^−^ at much lower concentrations as compared to other anions such as H_2_PO_4_^−^, I^−^, Br^−^, Cl^−^, F^−^, and NO_3_^−^ [79]. Free receptor showed two absorption bands at 291 nm (bpy-based π-π*) and at 462 nm (^3^MLCT band), which gradually decreased upon sequential addition of CH_3_COO^−^ to **42** along with a notable amplification of the absorption band at 346 nm with the onset of an isosbestic point at 376 nm and a new peak at 430 nm. Further, the free Ru(II) complex showed a strong emission band at 618 nm at room temperature upon excitation in the visible-light region (at 462 nm) in DMSO, which was subsequently quenched upon the addition of CH_3_COO^−^, F^−^ and H_2_PO_4_^−^. However, CH_3_COO^−^ was found to bring more spectral changes and turn off the luminescence behavior of **42**. Further mechanistic investigations pointed out that the triangular CH_3_COO^−^ (O-C-O angle of 120°) anion might be more favorable for the immediate binding to the imidazole and indole protons of **42** than tetrahedral anion such as H_2_PO_4_^−^ (since O-P-O angle of 108°). Thus, it was concluded that the distance of the oxygen atoms in CH_3_COO^−^ was accurate for bridging with the imidazole and indole protons, as shown in Scheme 2.

**Figure 7:**
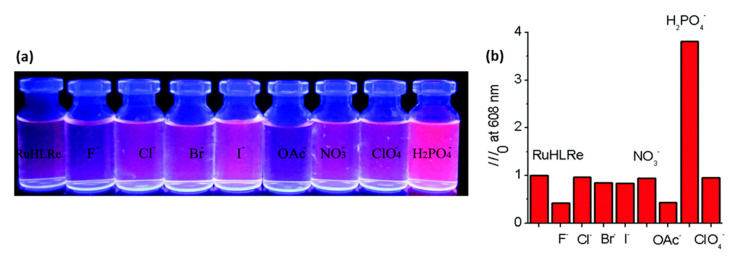
(**a**) Photographs of acetonitrile solutions of **44** (10μM) taken under a UV−lamp in the absence and presence of 10 equiv. of anions. (**b**) The PL emission intensity ratios of **44** were plotted in the presence and absence of the anions (λex = 460 nm). [Reproduced from reference [81]; Copyright © 2023, Royal Society of Chemistry].

Once more, this group systematically synthesized ruthenium (II) complex **43** and eventually prepared hetero bimetallic complex **44** (Figure 5) for the “turn-on” sensing of H_2_PO_4_^−^ and “turn-off” sensing of CH_3_COO^−^ and F^−^ (Figure 7) [80,81]. The Job’s plots analysis resulted in 1:1 host-to-guest stoichiometry for CH_3_COO^−^ and F^−^ while a 1:2 stoichiometry was reported for H_2_PO_4_^−^. The reason for 1:2 host-to-guest stoichiometry was attributed to the formation of two different types of hydrogen bonding by H_2_PO_4_^−^. One of the H_2_PO_4_^−^ ion formed an N–H⋯O hydrogen bond with the imidazole –NH, whereas the other formed a hydrogen bond with the pyridyl nitrogen atom of the receptor. Further, calculated binding constant values of **44** were 1.5 × 10^10^ M^−2^, 1.3 × 10^5^ M^−1^, and 5.5 × 10^4^ M^−1^ for H_2_PO_4_^−^, CH_3_COO^−^, and F^−^ respectively, which were comparatively greater than the calculated binding constant values for **43** (i.e., 7.6 × 10^9^ M^−2^, 3.7 × 10^4^ M^−1^, and 3.3 × 10^4^ M^−1^, for H_2_PO_4_^−^, CH_3_COO^−^, and F^−^ respectively). These binding constant results indicated **44** as a better sensor compared to its parent analog **43,** and it was well matched with the enhanced acidity of -NH of **44** (pKa 6.84 and 9.07 for **44** and **43,** respectively).

**Figure 8:**
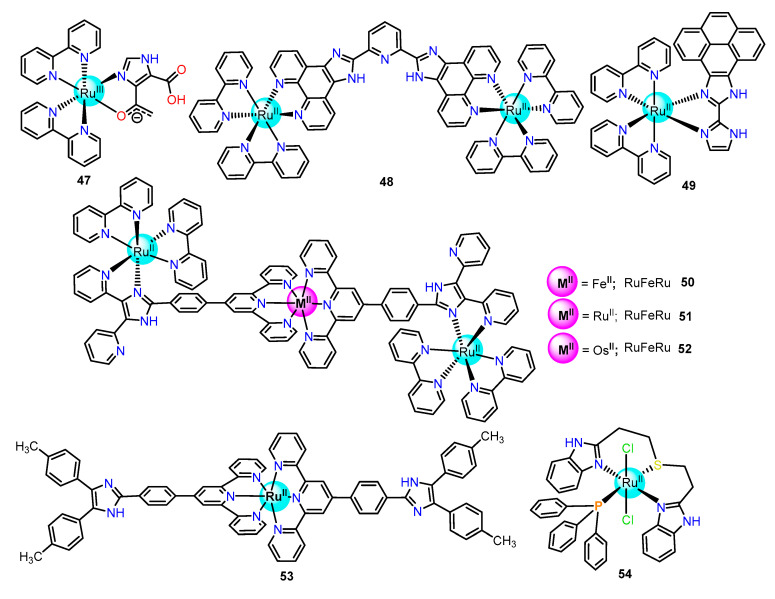
Structures of imidazole-based Ru(II) complexes **47**–**54**.

**Figure 9:**
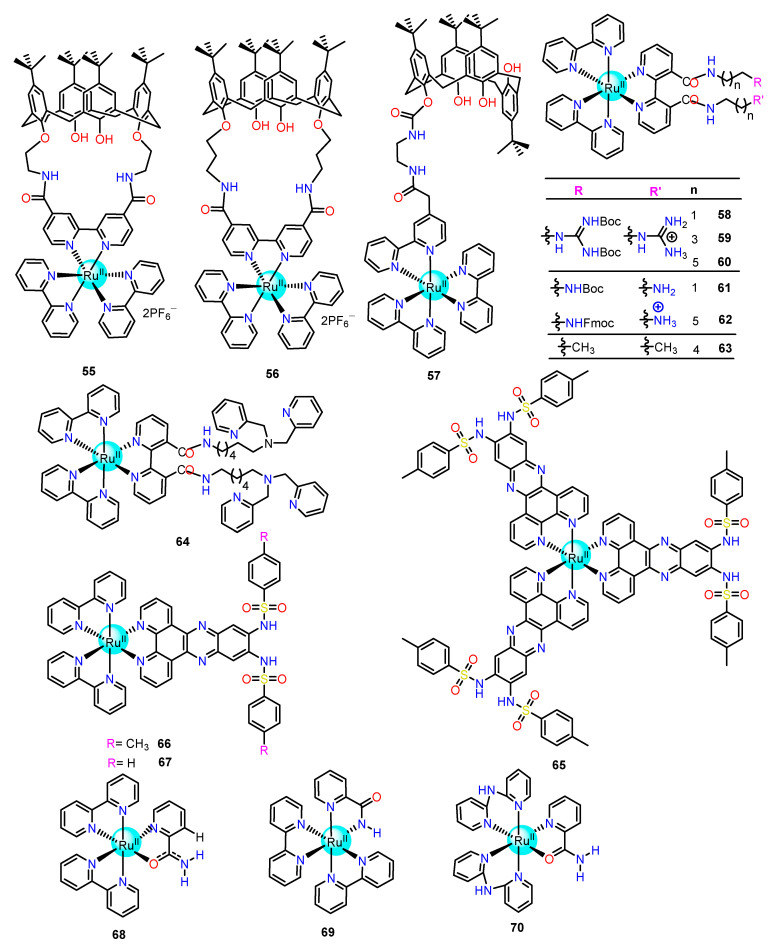
Structures of Amide/Sulphonamide/Picolinamide-based Ru(II) complexes **55**–**70**.

Further, Baitalik and his group developed monometallic and bimetallic ruthenium(II) complexes **45** and **46** (Figure 8) utilizing 4,5 bis(benzimidazol-2-yl)imidazole moiety as colorimetric sensors for anions [82]. Both complexes contained multipoint hydrogen bonds donating imidazole –NH protons for selective recognition and sensing of F^−^ and CH_3_COO^−^. It was shown that both **45** and **46** formed 1:1 hosts to guest adduct at lower concentrations of anions through hydrogen bonding interaction, while stepwise deprotonation of the imidazole –NH protons took place at higher concentrations. Later, the same group employed imidazole-4,5-dicarboxylic acid with Ru(II)-bipyridine moiety to synthesize **47** (Figure 9) as a luminescence anions sensor [83]. The photoluminescence intensity of **47** was increased upon the addition of F^−^, H_2_PO_4_^−^, and CH_3_COO^−^ ions. A significant red shift of emission maxima from 690 to 740 nm was observed, and a distinct color change of **47** was noticed in the presence of excess anions, which might be due to the deprotonation of imidazole –NH.

Again, second coordination sphere recognition of F^−^, H_2_PO_4_^−^, and CH_3_COO^−^ ions by bimetallic ruthenium (II) complexes **36** and **48** (Figure 8) containing acidic imidazole –NH protons were reported by the same group [84,85]. Remarkably, photo-physical experimental results displayed increased emission intensity as well as lifetime by H_2_PO_4_^−^ due to the rigidification of molecular backbone via hydrogen bonding interaction. However, a decrease in both was noted for F^−^ and CH_3_COO^−^caused due to the deprotonation of the imidazole -NH.

Pyrene-biimidazole-based Ru(II) chemosensor **49** (Figure 8) was developed by the same group for selective detection of CN^−^ in both acetonitrile and aqueous media [86]. The imidazole -NH protons of **49** were highly acidic, having pKa_1_ = 5.09 and pKa_2_ = 8.95. Deprotonation of these two -NHs by CN^–^ increased the electron density on the metal center. As a result, a red shift of absorption and quenching of emission of **49** was noted. The limit of detection of CN^−^ by **49** was 5.24 and 4.67 nM by the colorimetric and luminescent analyses methods, respectively.

Further, Baitalik et al. developed a trimetallic complex containing Fe(II) **50**, Ru(II) **51,** and Os(II) **52** (Figure 8) derived from a bipyridine-terpyridine heteroditopic type spanning ligand for sensing of selected anions in both organic and aqueous media [87]. All three triads acted as multichannel receptors for F^−^, CN^−^, H_2_PO_4_^−^, and CH_3_COO^−^ in acetonitrile without much selectivity. However, the triads displayed higher sensitivity and selectivity towards CN^−^ and SCN^−^ in water with a lower detection limit of 10^−8^ M. Similarly, recently developed Ru(II)-terpyridine complex **53** (Figure 8) was also selective for CN^−^ and capable of detecting CN^−^ ion in water up to the concentration limit 10^−8^ M [88]. 

Recently, Ramos and co-workers reported 1,5-bis(benzimidazol-2yl)-3-thiapentane-based ruthenium (II) complex **54** (Figure 8) for selective detection of Cl^−^ as a “switch on” probe even in the presence of other interfering anions such as F^−^, NO_3_^−^, Br^−^, HSO_4_^−^, I^−^, and H_2_PO_4_^−^ [89]. The interaction of Cl^−^ with the receptor complex facilitated the electron transfer from the highest occupied molecular orbital (HOMO) of the donor to low-lying HOMO of the fluorophore, i.e., acceptor as a disfavored Photo Electron Transfer (PET) process in the excited state resulted in the increasing the fluorescence intensity.

#### 2.1.3. Amide/Sulphonamide/Picolinamide Based Ru(II) Complexes as Chemosensors

Among the several polarized -NH functional groups, amide-based receptors are well known for sensing anions, while sulphonamide and picolinamide-based receptors are less explored. 

Through amendment of the [Ru(bpy)_3_]^2+^ with an amide containing t-butylcalix [4] arene moiety, Rajagopal et al. reported two new Ru(II) complexes **55** and **56** (Figure 9) for sensing of anions [90]. UV-Visible and PL emission studies revealed that **55** selectively recognized Cl^−^, H_2_PO_4_^−^, and CH_3_COO^−^, while **56** recognized Br^−^ and CH_3_COO^−^ anions. In both cases, the emission intensity was quenched by CH_3_COO^−^, where an increment of intensity was observed for other anions. Further, transient absorption and excited state lifetime studies were performed to find out the possible reasons for the quenching of emission in the case of acetate but enhancement for other anions. Through these studies, it was revealed that the excited states of both the receptors were stabilized by Cl^−^, Br^−^, and H_2_PO_4_^−^ anions which facilitated the radiative decay and instigated enhancement, while CH_3_COO^−^ destabilized the excited state through the non-radiative deactivation pathway and promoted quenching. 

**Scheme 3:**
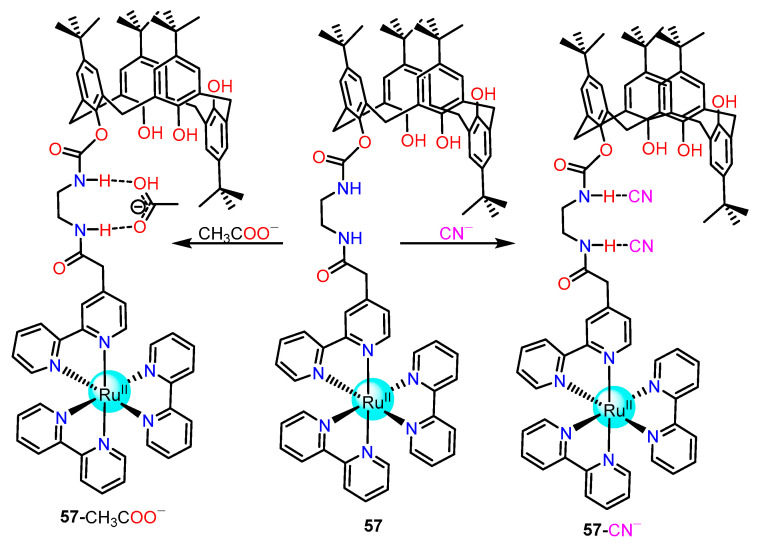
Modes of binding of **57** with CH_3_COO^−^ and CN^−^.

Another example of amide encompassing calixarene-based Ru(II) complex **57** (Figure 9) was developed by Maity et al. for selective recognition of CN^−^ and CH_3_COO^−^ [91]. The photo-luminescence studies of **57** in an aqueous organic medium (H_2_O:CH_3_CN 95:5) showed quenching for CN^−^, and enhancement of luminescence property was noted for CH_3_COO^−^. The mechanistic investigation exposed that CN^−^ resulted in deprotonation of amide –NH and increased the electron density in bipyridine ligand, and thus the intramolecular quenching was increased. On the contrary, bidentate CH_3_COO^−^ was bound with the amide –NH via weak hydrogen bonding interaction (Scheme 3). The hydrogen bounded CH_3_COO^−^ pulled the electron density and decreased the intramolecular quenching, thereby causing enhancement of luminescence intensity. The limit of detection value for CN^−^ calculated from luminescence response was 70 ppb.

Pinet et al. developed a series of modified 3,3′-bipyridyl-based novel ruthenium luminescent probes **58**–**64** (Figure 9) containing guanidinium, ammonium, or zinc(II) dipicolylamine binding sites for selective detection of anions in acetonitrile [92]. Hypsochromic shifts with enhanced luminescence intensity were found upon the addition of anions. Interestingly, guanidinium-functionalized probes were unveiled as more efficient sensors for CH_3_COO^−^. Receptor **60** showed higher selectivity towards glutamate preferentially to phosphates. Conversely, ammonium functionalized, **62,** selectively detects phosphates derivative. However, these probes were unable to bind such species in aqueous media due to the higher solvation energy of anions. 

Further, dipyridyl or phenanthroline moieties could be easily functionalized by the anion-sensitive bis(sulfonamide) group. Keeping this idea, Sun et al. developed Ru(II) complex **65** (Figure 9) comprising bis(sulfonamide) anion binding sites with highly chromophoric conjugated quinoxaline moieties for selective detection of F^−^ [93]. The Job’s plot analysis exposed a 1:1 host-guest complex for the receptor with F^−^. It was revealed that one sulfonamide interacts with the F^−^ while two other –NH protons is deprotonated in DMSO. It was assumed that the metalloreceptor **65** became a neutral molecule with two negative charges generated by the deprotonation of two –NH protons while only one was left to interact with the F^−^.

Besides, the Lin group reported sulfonamide-based ruthenium(II) complexes **66** and **67** (Figure 9) which displayed a strong affinity for F^−^ and CH_3_COO^−^ ions with adequate affinities for H_2_PO_4_^−^ or OH^−^ and almost no affinity for other halides [94]. Different photo-physical, electrochemical, and NMR studies revealed the anion binding abilities of Ru(II) complexes were in the order of CH_3_COO^−^ > F^−^ > H_2_PO_4_^−^≫ OH^−^ > Cl^−^, Br^−^, I^−^. Further, receptor **67** showed higher anion binding affinity over **66**. Thus, the relatively lower anion binding ability of the toluene-substituted complex **66** was ascribed to the better electron-donation of the methyl group compared to the hydrogen atom of the benzene analog. 

**Figure 10:**
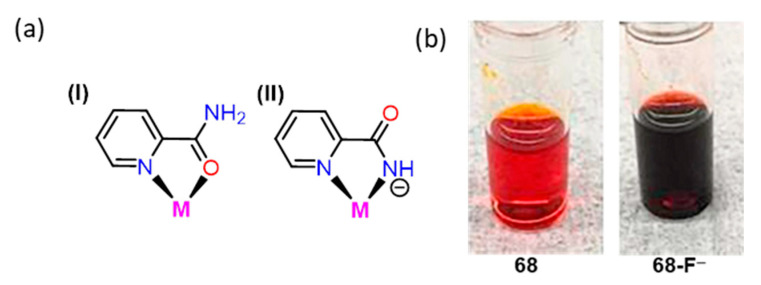
(**a**)Two coordination modes of 2-picolinamide: (I) H_2_pia-k^2^N,O (II) Hpia-k^2^N,N’; (**b**) The naked eye color of the solution of only **68** (left), and after adding TBAF (right). [Adapted from reference [91]; © 2023 Elsevier B.V. All rights reserved].

Again, neutral or deprotonated 2-picolinamide (i.e., H_2_pia and Hpia^−^, respectively) binds with many transition metal ions and has potential anticancer activity. It could bind with transition metal by two coordination modes: (I) pyridyl-N, and the deprotonated amide N jointly provided *N*, *N*’-coordination mode; (II) pyridyl N atom and the amide O provided the other *N*, *O*-coordination mode as shown in Figure 10a. However, the bis-heteroleptic ruthenium(II) complex with 2-picolinamide was scarcely used for the recognition and sensing of anions.

**Scheme 4:**
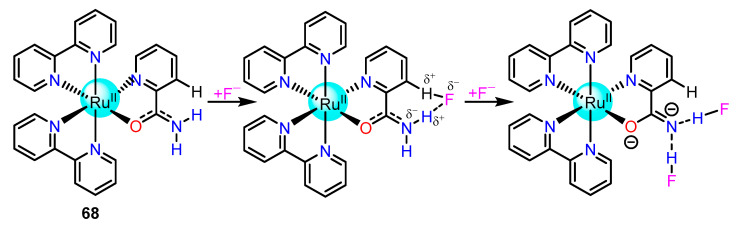
The stepwise reaction of **68** with F^−^ to give di-F^−^ adduct.

Endo group reported 2-picolinamide-based ruthenium complex **68** for naked-eye visual detection of F^−^ in acetonitrile [95]. Crystal structures of **68** revealed 2-picolinamide coordinated to Ru(II) via N,O coordination using N of the pyridyl group and O of the amide group. A visual color change from the red solution to dark-red was obtained upon the addition of F^−^ to **68** (Figure 10b). The technique ^1^H NMR and absorption spectroscopies exposed that the H near the pyridyl group of H_2_pia first formed a 1:1 adduct followed by the other H resulting in a 1:2 adduct as shown in Scheme 4.

**Scheme 5:**
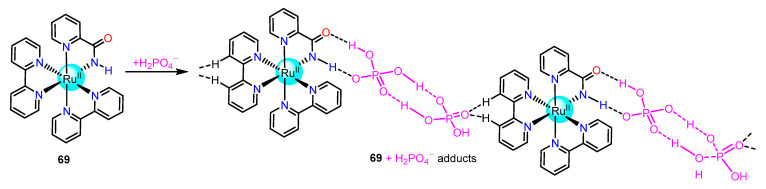
Schematic representation of the formation of the 1:2 adduct of **69** and H_2_PO_4_^−^.

Another interesting example of a 2-picolinamide-based Ru(II) complex **69** (Figure 9) was reported by Zhong and co-workers [96]. Single crystal X-ray characterization exhibited that the 2-picolinamide binds with Ru(II) center by the N atom of pyridyl and another N atom of the deprotonated amide group via N,N’-coordination mode. The spectroscopic and electrochemical studies revealed the receptor was selective for H_2_PO_4_^−^ over other common inorganic anions such as I^−^, Br^−^, Cl^−^, F^−^, NO_3_^−^, CH_3_COO^−^, HSO_4_^−^, and HP_2_O_7_^3−^. The Job’s plot analysis showed 1:2 hosts to guest binding with 10^8^ L^2^mol^−2^ global associations constant. The calculated limit of detection was 1.4 × 10^−6^ molL^−1^. Two wave behavior of Ru(II/III) redox couple of **69** was observed in the presence of H_2_PO_4_^−^ with a large negative shift from +0.74 to +0.45 V vs. Ag/AgCl. Again ^1^H NMR experiment exposed that a strong hydrogen bonding interaction between **69** and H_2_PO_4_^−^ plays a key role in the recognition process (Scheme 5).

Recently Nagao et al. reported 2-pycolinamide-based Ru (II) complex **70** (Figure 9) for selective colorimetric detection of F^−^ in DMSO [97]. Crystal structure analysis showed H_2_pia ligand and Ru(II) coordination via pyridyl-N and carbonyl-O, along with the carbonyl π-electron delocalized over the amide group. Further ^1^H NMR analysis and absorption spectroscopy showed that the added F^−^ did not differentiate amid the two amino groups of the di-2-pyridylamine and amide group of H_2_pia and thus formed a tris-F-adduct by turning the color from red to dark red. 

#### 2.1.4. Pyrrol-Based Ru(II) Complexes as Chemosensors

Among the various investigated anion receptors, pyrrol is one of the widely studied due to the presence of weakly acidic –NH proton (pKa of –NH proton is 16.5) that can form hydrogen bonding as well as become deprotonated by anions. Although, pyrrol or substituted pyrrol-based ruthenium (II) complex are scarcely reported in the literature as an anion sensor.

**Figure 11:**
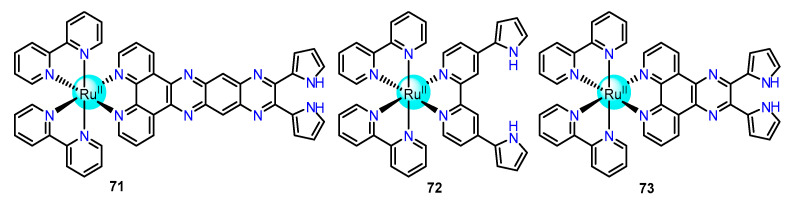
Structures of dipyrrol-based Ru(II) complexes **71**–**73**.

Fused dipyrrolylquinoxaline (DPQ) phenanthroline-based ruthenium(II) complex **71** (Figure 11) was designed and synthesized by Sessler and his group for selective detection of F^−^ in DMSO [98]. The acidic nature of the pyrrol –NH protons were increased upon binding the DPQ-phenanthroline ligand with the Ru(II) metal center. Further, Job’s plot analysis showed a 1:1 hosts-to-guest binding. The calculated binding constant was 1.2 × 10^4^ M^−1^, which is nearly 30 times greater than that calculated from the free ligand. 

Thereafter the same group replaced the quinoxaline with a bipyridine moiety for the generation of a larger binding site and to achieve enhanced binding affinity towards larger anions. In this regard, dipyrrol-appended bipyridine-based Ru(II) complex **72** (Figure 11) was synthesized for emission-based selective sensing of H_2_PO_4_^−^ [99]. Emission intensity at 630 nm of the receptor was decreased upon the addition of anions. The Job’s plot analysis resulted in 1:1 hosts to guest binding and the binding constant value for H_2_PO_4_^−^ was 1.0 × 10^5^ M^−1^. 

The development of time-resolved fluorescence method-based chemical sensing of anion is advantageous since the lifetime is independent of the excitation source, intensity fluctuations, total probe intensity, loss of light in the optical path, and sensitivity of the detector. Anzenbacher’s group first reported lifetime-based anion sensing by Ru(II) complex, **73** (Figure 11) having DPQ-phenanthroline as an anion coordinating site [100]. Photophysical studies showed a decrease in the absorption band corresponding to MLCT and the high-energy π-π* ligand in addition to quenching of luminescence intensity upon the addition of CN^−^ and F^−^. The binding constants for CN^−^ and F^−^ were found to be 4.3 × 10^5^ M^−1^ and 6.4 × 10^5^ M^−1^, respectively, in acetonitrile solutions. Further luminescence lifetime of **73** was shortened minutely from 377 ± 20 ns to 341 ± 20 ns upon the addition of 6.20 µM of CN^−^. Two lifetimes shortening was obtained ranging from 13 to 17 ns (short τ) and 320 to 370 ns (long τ) upon excessive addition of CN^−^ which was fitted in two exponential decay fitting equations.

#### 2.1.5. Urea-Based Ru(II) Complexes as Chemosensors

Urea is mostly explored as a classical neutral anion receptor as it is capable of forming hydrogen bonding interactions with anions. Most of the halide could interact with urea through the formation of a six-membered chelate ring, whereas oxyanions (having nearby two oxygen) could form eight-membered chelate rings. In the last two decades, Wilcox [101] and Hamilton [102] synthesized an array of receptors comprising one or more urea entities aiming to control the substituents choice and the polarization of the –NH units. 

**Figure 12:**
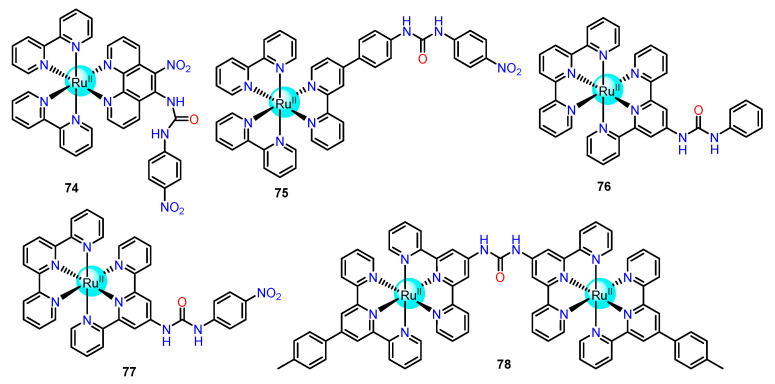
Structures of urea-based Ru(II) complexes **74**–**78**.

Das et al. reported urea-based Ru(II) complex **74** (Figure 12) that acted as a colorimetric sensor for basic anions such as F^−^, CH_3_COO^−^, and H_2_PO_4_^−^ [103]. Different spectroscopic techniques, including ^1^H NMR titration studies, concluded that the ligand is bound with H_2_PO_4_^−^, F^−^, and CH_3_COO^−^ more strongly than the other halides and oxyanions. Further photoluminescence studies showed the emission intensity of **74** is completely quenched by F^−^, CH_3_COO^−^, and H_2_PO_4_^−^. It is because of the faster decay of the excited triplet state by reducing the energy gap (excited triplet state and ground singlet states) upon anion binding that increased the solvation of the anionic adducts or deprotonated state of **74**.

Ru(II)-polypyridyl complex **75** (Figure 12), having pendent urea moiety as an anion recognition unit, was synthesized by Gunnlaugsson and co-workers, which worked as a long-wavelength fluorescent sensor for anions [104]. The ^1^H NMR titrations demonstrated the interaction between the urea moiety and anion via hydrogen bonding interaction which played an important role in the recognition process. Further, MLCT emission is sensitive towards the binding of CH_3_COO^−^, H_2_PO_4_^−^, and HP_2_O_7_^3−^ but not for F^−^ in the organic solvent. Remarkably **75** could differentiate between phosphate and pyrophosphate through “turn on’’ emission in the presence of H_2_PO_4_^−^ and ‘‘turn-off’’ emission in the presence of HP_2_O_7_^3−^. 

Again, sensing and recognition properties of the urea subunit toward anions could be improved by linking with Ru(terpy)_2_^2+^ instead of Ru(bpy)_3_^2+^ units. Advantages of the use of Ru(terpy)_2_^2+^ appeared to result from (i) a through-space electrostatic effect and (ii) a through-bond covalent effect. Further, a combination effect of Ru(II) polypyridine-metal complex and pendent substituents could enhance the anion binding affinity. In this regard, Fabbrizzi et al. developed terpyridine-based Ru(II) complex **76** and improved the acidity of the urea unit by substituting pendent benzene with nitrobenzene **77** (Figure 12) [105]. Hydrogen bonding interaction between urea proton and anions resulted in a 1:1 stoichiometry receptor-anion complex which was confirmed by Job’s plot. The log K values for the binding of Cl^−^ were 5.66 ± 0.01 and 6.32 ± 0.02, corresponding to **76** and **77**, respectively. The binding affinity decreased across the series Cl^−^ > Br^−^ > I^−^, which was parallel to the decreased charge density of the anion. 

Yao and co-workers showed how the interaction of anion and urea could be engaged to control the electron transfer and electronic coupling between redox-active sites [106]. For this purpose, this group synthesized urea-functionalized diruthenium complex **78** (Figure 12). A detail electrochemical study along with DFT suggested that the electronic coupling between two cyclometalated ruthenium and urea might enhance the degree of binding of Cl^−^ or Br^−^ ions to urea units via hydrogen bonding interactions. Further, the redox wave reversibility of **78** was fully maintained in the presence of Br^−^ or Cl^−^. However, strong basic anions, for example, F^−^, CH_3_COO^−^, and H_2_PO_4_^−^ ruined the redox waves by making them highly irreversible. 

#### 2.1.6. Aldehyde Incorporated Ru(II) Complexes as Chemodosimeters

Chemodosimeter is a term first described by Chae and Czarnik [107]. It is an abiotic molecule achieved by analyte recognition through observable signal output. This approach involves the reaction of a molecular receptor, known as a chemidosimeter, with the analyte followed by substantial chemical transformation, including breaking and forming of the covalent bonds. 

**Figure 13:**
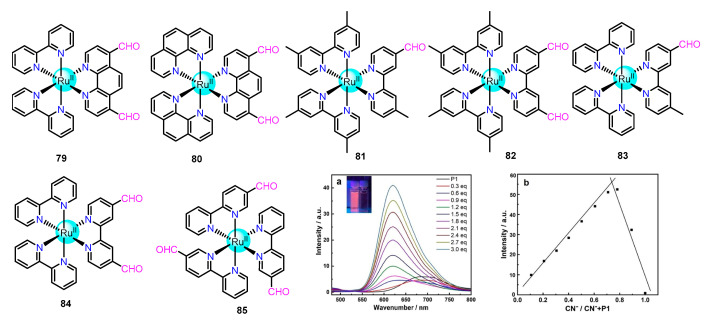
Structures of Aldehyde group containing Ru(II) complexes **81**–**84**. (**a**) phosphorescence titration of **85** with CN^−^ (0.3 eq/time) (inset left: **85**, right: **85** + 3CN^–^). (**b**) Job′s plots of CN^−^ adduct complexes of 85. [Adapted from reference [109]; Copyright © 2023, Royal Society of Chemistry].

Schmittel’s group presented two new ruthenium complexes **79** and **80** (Figure 13) with 1,10-phenanthroline-4,7-dicarboxaldehyde (PDA) as a chelating ligand to detect CN^−^ based on the cyanohydrin’s formation [108]. The immediate change of color (orange-red to yellow) was detected through the naked eye after CN^−^ addition. Further, PL studies displayed a drastic blue shift of almost 100 nm and ~55-fold enhancement of emission intensity within 15 s in the presence of 2 equiv. of CN^−^ to both the receptor’s solutions. A large value of overall cyanohydrin formation constants was obtained from PL titration for the two receptors (log β_[CN_^−^_]_ = 15.36 ± 0.44 and log β_[CN_^−^_]_ = 16.37 ± 0.53 for **79** and **80,** respectively).

Later Chen et al. reported four ruthenium(II) complexes **81**–**84** (Figure 13) which selectively sensed CN^−^ by the formation of a well-known cyanohydrin complex [110]. All these complexes showed similar types of photo-physical properties. A visible naked-eye change of color (orange to yellow), along with a fluorescent distinction (dark red to red-orange), was perceived after the addition of CN^−^ to the Ru(II) complex solution. Further, a large blue shift of the absorption and emission spectra with a notable increment in the emission was observed after the addition of CN^−^

Again, Yao et al. reported 5-aldehyde-2,20- bipyridine-based Ru(II) complex **85** (Figure 13) for selective detection of CN^−^ in an aqueous acetonitrile solution (60% water) [109]. Similar to the previous receptors, it was highly selective and sensitive toward CN^−^. A visible color change (orange-red to yellow) was observed. It worked as a phosphorescence “turn-on” response probe with a ~30- fold increase in emission intensity for the formation of cyanohydrin through nucleophilic addition of CN^−^ to aldehyde (Figure 13a). The formation of cyanohydrin followed the 1:3 stoichiometry of host to guest, and a detection limit corresponding to 0.75 μM was calculated(Figure 13b).

#### 2.1.7. Some Example of Bis-Heteroleptic Ru(II) Complexes as Chemosensor

In this category, we give some examples of bis-heteroleptic Ru(II) complexes that could act as chemosensors for different anions.

**Figure 14:**
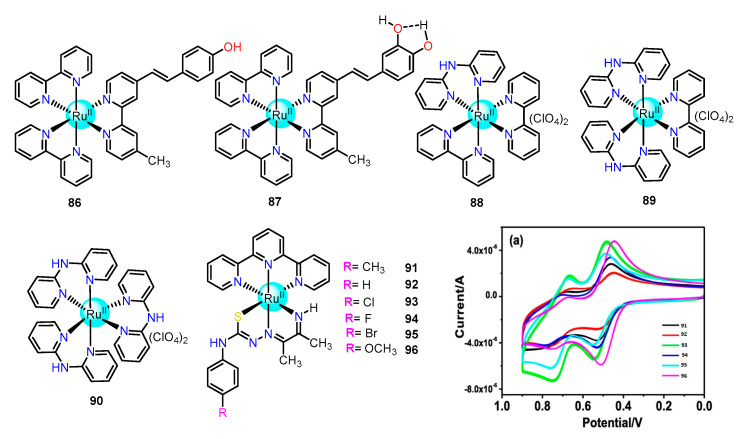
Structures of 2,2’-dipyridylamine−based Ru(II) complexes **88**–**96** and (**a**) cyclic voltammograms of **91**–**96,** recorded in acetonitrile solution with Ag/AgCl/KCl(std.) electrode. [Adapted from reference [112]; © 2023 Elsevier B.V. All rights reserved].

**Scheme 6:**
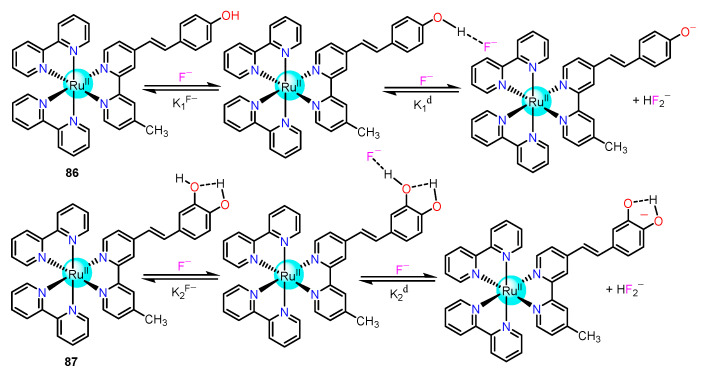
Mechanism of deprotonation of **86** and **87** via hydrogen bonding interaction.

In 2007, Das et al. reported phenol and catechol-based Ru(II) polypyridile complexes **86** and **87** (Figure 14), which acted as efficient colorimetric sensors for F^−^ even in the presence of ~20% H_2_O (*v*/*v*) [111]. Different spectroscopic techniques and time-dependent density-functional theory (TDDFT) revealed that the hydrogen bonding interaction between F^−^ and O-H of phenol or catechol occurred at lower concentrations while higher concentrations of anions promoted deprotonation of O-H (Scheme 6).

Das and co-workers developed three mononuclear Ru(II) complexes **88**–**90** (Figure 14) comprising 2,2’-bipyridine and 2,2’-dipyridylamine for selective detection of F^−^ and CN^−^ [113]. The spectrophotometry, electrochemistry, and ^1^H NMR spectroscopy studies revealed that all these complexes were selective for F^−^ and CN^−^ over other common inorganic anions (for example, PF_6_^−^, Br^−^, Cl^−^, ClO_4_^−^, NO_3_^−^, HSO_4_^−^, and CH_3_COO^−^). The recognition and sensing phenomena occurred through the formation of sequentially deprotonated complexes for all three receptors, a hydrogen-bonded refereed adduct. The stoichiometry of binding was 1:1, 1:2, and 1:3, with **88**, **89**, and **90**, respectively, for CN^−^ and F^−^. Again, Chattopadhyay et al. developed six thiosemicarbazones containing Ru(II) complexes **91**–**96** (Figure 14) for sensing F^−^ [112]. Cyclic voltammetric (CV) measurement showed all the complexes underwent two quasi-reversible oxidations on the positive side of the potential window (0 to +0.8 V) and three consecutive quasi-reversible/irreversible reductions on the negative side of the potential window (0 to 2 V) (Figure 14a). Further, it was found that the binding constants were practically high (logKa > 5) for complexes **93**–**96**. 

**Scheme 7:**
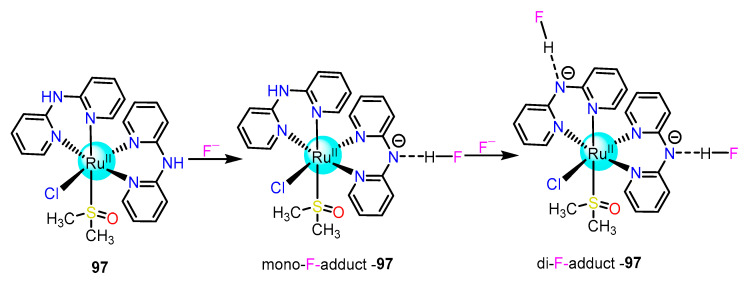
Formation of mono and di-F^−^ adduct of **97**.

Nagao et al. reported C_1_-symmetricRu(II) complex **97**, where two distinguishable –NH groups acted as Cl^−^ and F^−^ receptors via hydrogen bonding interactions [114]. The use of ^1^H NMR demonstrated that the–NH of di-2-pyridylamine formed selectively and successively mono F^−^ adduct followed by di-F^−^ adduct of **97** upon addition of F^−^ to the receptor solution in DMSO (Scheme 7).

### 2.2. Cyclometalated Iridium(III)-Based Complexes for Sensing of Anions

Complexes of iridium usually exhibit a high room temperature quantum efficiency, high thermal and electrochemical stabilities, good photo-stability, and large Stokes shift, which restricts self-quenching, readily tunable emission wavelengths (green to red), which could easily be altered by changing the nature of the secondary co-ligands. Owing to their ease of synthesis and their excellent photo-physical and electrochemical properties, cyclometalated iridium complexes have been receiving a lot of attention for their application as molecular sensors. This section of the review highlights the application of luminescent iridium complexes for sensing and recognition of anions.

#### 2.2.1. Fluoride Sensing

It is widely known that fluoride anions (F^−^) play vital roles in an extensive range of chemical, biological, and environmental processes. Medical practices such as the treatment of osteoporosis and those for dental care, environmental treatments such as fluorination of water supplies, and the use of F^−^ in chemical and nuclear warfare agents have resulted in increased human exposure [115]. High doses of F^−^ are, however, detrimental and can lead to dental or skeletal fluorosis. Therefore, the recognition and detection of this anion is an active area of research, and a great deal of effort has been devoted to the design of molecular receptors containing binding sites for fluoride.

Receptors based on Lewis acid-base interactions between boron and F^−^ were developed and established to accomplish highly efficient detection. In particular, sterically hindered boryl groups, for instance, dimesitylboryl moieties, have shown amended size-selectivity toward fluoride ions. The strong B–F interaction could hold up the π-conjugation of organoboron compounds, thus resulting in changes in absorption colorimetry, fluorescence emission, spectral shifts in NMR, and electrochemical properties. In this regard, numerous cyclometalated Ir(III) complexes conjugated with organoborane have drawn attention for their abilities as colorimetric chemosensors selective for fluoride anion.

**Figure 15:**
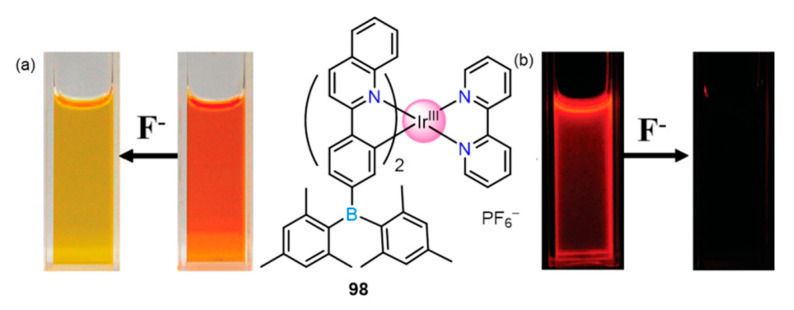
Structure of Ir(III) complex **98** and (**a**) naked eye color change; (**b**) emission color observed under a UV lamp in the absence and presence of F^−^. [Adapted from reference [116]; Copyright © 2023, American Chemical Society].

A naked eye chemosensor of F^−^ via a phosphorescent iridium complex **98**, functionalized with an arylborane unit, was reported by Huang et al. A visible color change from yellow to reddish-orange was noticed in the presence of 3 equiv. of F^−^ anion with the subsequent red shifting (420−600 nm) of the absorption band (Figure 15) [116]. This shift was caused by the complexation of fluoride to cyclometalated arylborane ligand, which resulted in the excited-state switch from π-π* to CT transition of the adduct. The binding constant from UV-Vis titration data was put to 1:2 binding isotherm for complex-fluoride adduct, which awarded the K_1_ = 1.29 × 10^6^ and K_2_ = 4.27 × 10^5^ M^−1^. The photo-luminescence emission spectra showed a quenching phenomenon upon the 2 equiv. addition of F^−^ anion, thus making it an “ON−OFF” type probe. 

**Figure 16:**
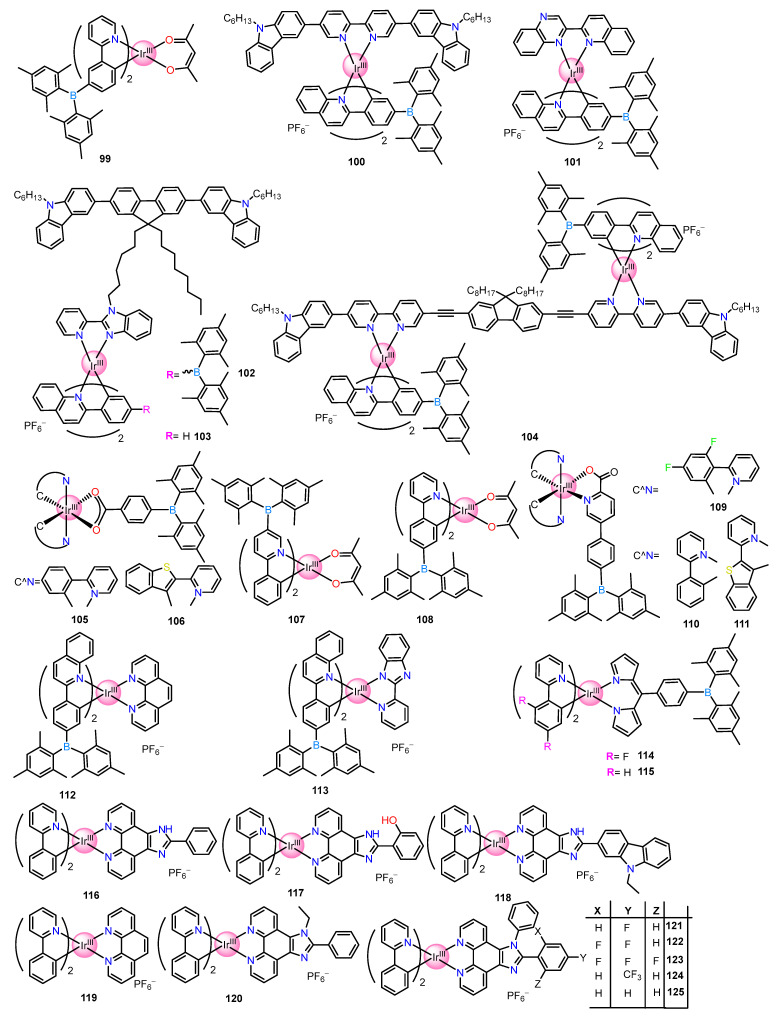
Structures of Ir(III) complexes **99**–**125**.

Again, the Park group in 2008 designed and synthesized a sterically congested dimesityboryl group-based phenylpyridine ligand and complexed it with an iridium center to produce a highly phosphorescent complex **99** (Figure 16), which served as a fluoride sensor via colorimetric and ratiometric responses [117]. The complex showed not only high selectivity for the fluoride ion but also an efficient two-color phosphorescence behavior. In addition, a high signal-to-noise ratio was reached through the time-gated acquisition of phosphorescence signals. Furthermore, successful aqueous medium detection of fluoride was realized by doping the iridium complex with PMMA. 

A novel cationic iridium (III) complex **100** (Figure 16), containing carbazole and dimesitylboryl moieties, was synthesized by Huan et al. for colorimetric and ratiometric sensing of F^−^ [118]. A quenching in the orange-red phosphorescent emission of the complex was observed upon fluoride binding along with the switch on of fluorescent emission from the N^N ligand, which is reflected by a visual change in the emission color from orange-red to blue. The mechanistic studies of the fluoride adduct were conducted through TDDFT, which revealed that the free complex has a triplet emission state and the energy transfer occurred from the carbazolyl bipyridine moiety to the iridium (III) center. However, upon the interaction of F^−^, the emission took place from the singlet excited state, and thus the energy transfer was inhibited. Hence the complex displayed fluoride sensing/interaction via a triplet to singlet switch-on behavior. Only fluoride was able to perturb the emission spectra of the complex, while the spectra remained unchanged in the presence of other anions. A low detection limit corresponding to the range 0–50 um was calculated with F^−^ anion. 

The same group synthesized complex **101** (Figure 16)**,** which was a near-infrared (NIR) phosphorescent chemosensor of F^−^ containing dimesitylboryl (Mes_2_B) groups and an iridium (III) center [119]. The selective binding of F^−^ caused quenching of the NIR phosphorescent emission with λ_em_ of 680 nm exhibited by the complex. From the emission titration data, the binding constants K_1_ and K_2_ were found to be 2.64 × 10^6^ and 2.12 × 10^4^ M^−1^, respectively.

Later this group developed a Förster (Fluorescence) Resonance Energy Transfer (FRET)-based F^−^ probe, **102** (Figure 16), comprising of Mes_2_B unit-functionalized with cationic Ir(III) complex as phosphorescent acceptor unit and carbazole-fluorene-carbazole as the fluorescent donor unit [120]. In addition, the group synthesized **103**, which was similar to **102**, but without the Mes_2_B group. The inception of Mes_2_B groups into the ligand part of the Ir(III) complex leads to red-shifting along with strong absorption and phosphorescence. Further, the efficacy of the FRET process from the donor of fluorescent to the acceptor of phosphorescent was increased significantly due to the Mes_2_B groups. The excited-state properties of Ir(III) perturbed and suppressed the FRET process from donor to acceptor upon binding of F^−^, which improved the blue emission from the fluorescent donor. So, **102** could be used as switchable phosphorescence and fluorescence sensor due to its higher selectivity towards F^−^. 

Again, a new D-A-π-A-D dinuclear phosphorescent complex of iridium, **104** (Figure 16), containing dimesitylboryl groups on the cyclometalated C^N ligands (Bpq) was developed by Huang and co-workers by modification of **98 [121].** The introduction of extended conjugation in the ligand backbone made **104** a highly effective orange-red phosphorescent emitter (emission wavelength of 606 nm) and displayed a quantum efficiency of 0.13 at room temperature. Further, complex **104** showed two photon-absorption cross-sections having a maximum value of 481 GM which was much higher than the non-metallated ligand as well as from the mononuclear **98**. Upon checking the complexing ability of **104** with F^−^, it was observed that the absorption bands gradually decreased, and blue shifting was noted along with three isosbestic points. In addition, the SPEF and OPEP emission spectra showed quenching upon the addition of increasing concentration of F^−^ to **104,** and thus it can be used as an “ON-OFF” switch probe for F^−^.

**Scheme 8:**
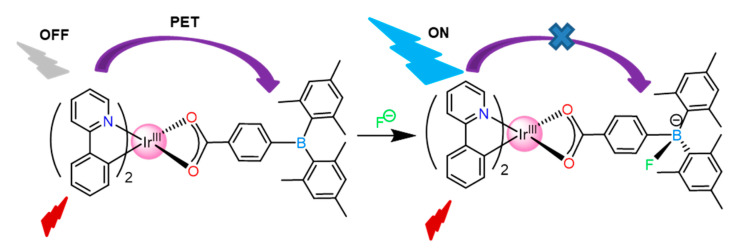
Mechanism of PET process of **105** with “OFF-ON” F^−^ sensor.

Bis-heterolptic iridium (III) complexes **105** and **106** (Figure 16), having 4-(dimesitylboryl)benzoate as an auxiliary ligand, were reported by Lee and his group for a unique phosphorescence turn-on response in the presence of F^−^ [122]. It was proposed through experimental and DFT calculations that the complex originally exhibited a weak PL emission due to the forward PET from the iridium ppy center to the 4-(dimesitylboryl)benzoate, which subsequently is inhibited upon F^−^ binding and thus resulting in switching on the ^3^MLCT phosphorescence with a consequent increase in the intensity of the band (Scheme 8). A high binding constant corresponding to 8.0 × 10^6^ M^−1^ and 9.0 × 10^6^ M^−1^ for **105** and **106,** respectively, was assessed from the UV−Vis titrations experiments when fitted in the 1:1 binding model.

Wong and group incorporated organoborane moiety into ppy ligand and complexed it with Ir(III) metal to give isomeric complexes **107** and **108** (Figure 16), which gave intense red phosphorescence [123]. In **107** and **108,** the p-orbital of B(Mes)_2_ contributes to the lowest unoccupied molecular orbital (LUMO), which is traditionally different from the typical Ir-ppy complexes where the p-orbital on the pyridyl unit contributes predominantly. This caused a shift in the electron transfer in the MLCT process from pyridyl units to the B(Mes)_2_ moieties and induced stabilized low-energy ^3^MLCT states in both complexes. In the case of **107**, the color changed from red to yellow, accompanied by a new band at 556 nm, probably caused by destabilizing the MLCT state owing to the repulsion interaction between the electron-rich boron center and F^−^. In the second step, the addition of more F^−^ generated a new green phosphorescent band at 505 nm via a strong binding between F^−^ and the boron center, as the repulsion interaction between them is reduced due to the electron density transfer from the boron center to pyridyl unit. The above example demonstrated a reversible, highly sensitive, and selective chemosensor for F^−^.

Lee et al. reported borane conjugated heteroleptic (C^N)_2_Ir^III^ complexes **109**–**111** (Figure 16), assisted by ancillary triarylborylpicolinate (Bpic) ligand as immensely sensitive, ratiometric and/or turn-on phosphorescence sensors for F^−^ [124]. Experimental and theoretical studies revealed the LUMO of **109** and **110** were mostly localized over Bpic ligand, which made weakly emissive ^3^ML′CT/^3^LL′CT (L = C^N; L′ = Bpic) states as the lowest-energy triplet excited state. However, binding of F^−^ to these probes induced the highly emissive ^3^MLCT/^3^ππ* (L = C^N) states which were centered on the (C^N)_2_Ir^III^ moiety. Consequently, **109** and **110** became ratiometric turn-on phosphorescence sensors for F^−^, while the phosphorescence property of **111** remained notably unaffected.

Utilizing molecules having optoelectronic properties as molecular switches have fascinated increasing attention for their applications in molecular photonic and electronic devices. It is observed that external physical and chemical stimuli can bring about fast and reversible responses in these molecular switches. In this regard, Huang and coworkers reported two new complexes of iridium **112** and **113** (Figure 16), possessing bulky dimesitylboryl (Mes_2_B) moieties, which prevented the boron center from nonspecific nucleophilic attack [125]. The complex in acetonitrile exhibited an orange-red wide emission band centered at 595 nm with a shoulder at 637 nm. Both complexes showed a quenching phenomenon upon F^−^ addition through the formation of B–F bonds. Further, DFT calculations were performed to understand the reason for the quenching behaviors, which showed that the HOMO of complex **112** is localized on the iridium atom and dimesitylboryl (Mes_2_B) moieties while (LUMO) resides on the N^N ligand. Thus, the phosphorescence in the complex originated from ^3^MLCT and ^3^LLCT transitions. Whereas for the fluoride adduct, the HOMO comes from the Mes_2_B–F^−^ fragments, and the LUMO resides on the N^N ligand hence the lowest triplet state originates from the HOMO/LUMO transition. Thus, upon complexation, the excited-state properties of the complex are changed, resulting in emission quenching. Interestingly, the quenched phosphoresce reappeared near the anode, and it is supposed that the electric field caused the rupture of the B–F bond, and hence the red-orange phosphorescence remerged. Thus, the complex acts as a reversible phosphorescence optoelectronic device by using the fluoride and electric field, and thus an INH logic gate is constructed by using F^−^ and an electric field.

Lee et al. reported two novel borane-coupled Ir(III)-complexes **114** and **115** (Figure 16), chelated by a dipyrromethene-based ancillary ligand as ratiometrically “turn-on” emissive probes for F^−^ in the near IR region. Both these receptors were well characterized by different spectroscopic and solid-state single-crystal X-ray diffraction measurements [126]. Further UV-Vis studies showed the typical ^1^MLCT absorption bands at ~483 nm, while with CT absorption band corresponding to the borane center appeared at ~326 nm. From the PL studies, the emissions were observed at ~675 nm corresponding to the ^3^MLCT emission. Further, the F^−^ titration experiment showed gradual blue-shifting of the emission spectra with corresponding Δλ values of 27 nm and 34 nm for **114** and **115**, respectively, accompanied by a ratiometric turn-on of the emissive properties. The turn-on emissive response by **114** and **115** were established through theoretical calculations.

Amongst various heterocyclic units, the imidazole ring provided excellent hydrogen bond donor sites for anion binding. The acidity of the –NH proton in the imidazole ring can easily be modified by functionalizing the imidazole unit with different substituents having varied electronic properties. Tailoring the imidazole unit with a ligand having a donor pyridine-like nitrogen atom capable of complexing with phosphorescent metal centers converted the imidazole derivatives into excellent luminophores for anion binding.

Huang group reported five cationic iridium salts (**116**–**120**) (Figure 16). Among them, the three complexes **116**–**118** have distinctly substituted phenanthroline-imidazole units, with each containing anion binding –NH site, while **119** contains just the phenanthroline and **120** has imidazole phenanthroline moiety but lacks the –NH unit [127]. Photophysical and electrochemical studies showed **116**–**118** were selective towards basic anions such as F^−^, H_2_PO_4_^−^, and CH_3_COO^−^. Furthermore, the addition of these three basic anions to the DCM solution of **116**–**118** showed an impressive naked-eye color change from yellow-green to brown. All three complexes showed intense luminescence emission in the range of 568–583 nm, and the addition of F^−^ to all these complexes resulted in phosphoresce quenching, probably caused by the PET from the lone pair of imidazolyl group after deprotonation. A similar change in absorbance and emission spectra was noted for these complexes upon the addition of H_2_PO_4_^−^ and CH_3_COO^−^, whereas other anions failed to show a similar change. Calculation of the binding constant in each case revealed that all three complexes prefer to bind F^−^ anion over H_2_PO_4_^−^ and CH_3_COO^−^. The extent of binding is preferably more for complex **116,** and hence it can be concluded that **116** can act as an ideal phosphorescent probe for F^−^.

A series of heteroleptic cationic salts of iridium having different phenanthroline derivatives with supremely electron retreating fluoro- and trifluoromethyl- substituents at preferred positions were developed, and their effects on the photophysical and electrochemical properties by anions were studied [128]. The complexing capabilities of **121**–**125** (Figure 16) with different anions were probed through UV–Vis titration experimentation. These complexes showed a strong absorption band of ~263 nm and are allocated to spin-allowed π-π* transitions of phenanthroline ligands. The presence of F^−^ influenced the band at 263 nm to decrease gradually, accompanied by the appearance of new bands at 308, 442, and 478 nm, along with three different isosbestic points. A change in color from green to brown is also observed for **121**. Complexes **122**–**124** reported a similar change upon F^−^ addition. The complexes emit in the range of 564–582 nm in DCM solution, and the titration profile of all the complexes resulted in a quenching phenomenon. Interestingly, a similar result was obtained upon the addition of CH_3_COO^−^ and H_2_PO_4_^−^. A binding constant of 4.92×10^3^, 2.63×10^4^, and 6.98× 10^4^ M^−1^ for H_2_PO_4_^−^, CH_3_COO^−^, and F^−^, respectively were reported for **121**. Further, it was concluded through a controlled experiment that the considerable optical response was due to the interaction of –NH of phenanthroline ligands with the anions. Additionally, the enhanced acidity of –NH and deprotonation trend was caused by the intramolecular N-H-F-P hydrogen bonds existing in these complexes.

**Figure 17:**
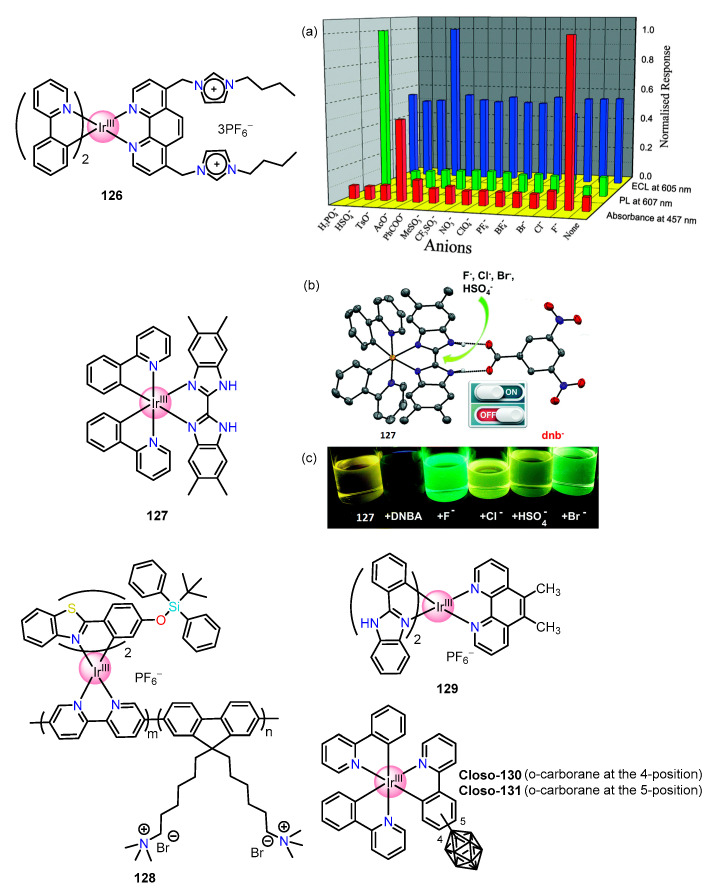
Structures of Ir(III) complexes **126**–**131**; (**a**)The comparative response of complex **126** in UV−Vis, PL, and ECL upon the addition of anions. [Adapted from reference [129]; Copyright © 2023, Royal Society of Chemistry]. (**b**) luminescent on/off sensor for anions; (**c**) picture of **127** in chloroform and excess anions. [adapted from reference [130]; Copyright © 2023, Royal Society of Chemistry].

A competitive iridium-based quantification of three different anions in a mixture via three different interrogation techniques and depending upon the anion-imidazolium interaction was reported by Schmittel et al. [129]. The following anions H_2_PO_4_^−^, HSO_4_^−^, F^−^, Cl^−^, Br^−^, PhCOO^−^, BF_4_^−^, PF_6_^−^, ClO_4_^−^, NO_3_^−^, CF_3_SO_3_^−^, MeSO_3_^−^, CH_3_CO_2_^−^, and TsO^−^ were tested in three different channels (Figure 17a). The addition of F^−^ to **126** caused a broadening and increase in absorption intensity at 457 nm, indicating a new electron-donating unit, whereas the addition of CH_3_CO_2_^−^ caused a slight hyperchromic shift, whereas other anions did not induce any such similar changes. The ^1^H NMR analysis of complex **126** showed a downfield shift corresponding to Δδ = 0.62 ppm, indicating an ionic hydrogen-fluoride bond accompanied by H/D exchange of both 2-H and the methylene protons of **126** in a mixture of CD_3_CN/CD_3_OD (*v*/*v*, 9:1). The Job’s plot was fitted in a 1:2 binding model for UV-titration results which gave the binding constant as log b = 9.71 ± 0.30 and the limit of detection was calculated as 0.21 μM in the presence of F^−^. Complex **126** emits at 660 nm, and the emission shifted to 607 nm with 7-fold increases in emission intensity upon the addition of 5 equiv. of H_2_PO_4_^−^.From the PL and UV titration profile, the binding constant was calculated to be logβ = 7.18 ± 0.10, and the corresponding detection limit was 68 nM. Here the NMR showed a slight downfield shift in the imidazolium proton. In oxidative ECL scans, emission at 605 nm was displayed by complex **126**. A highly selective ECL enhancement was shown by **126** in the presence of CH_3_CO_2_^−^ while other anions failed to perturb the result. Here the limit of detection was calculated to be 0.17 mM. It is proposed from the results that the positively charged iridium center and the imidazolium units form a tricationic cavity that serves to bind a single CH_3_CO_2_^−^ in a bifurcated hydrogen bond. Thus, three different anions were quantified using three techniques by receptor **126**.

In 2016, Rau reported a cis-diamine bibenzimidazole functionalized iridium receptor **127** (Figure 17) as an anion sensor [131]. The hydrogen bonding interaction between complex **127** and the anion influenced its photophysical properties. Titration with fluoride caused blue sifting and an increase in emission intensity until 1 equiv. of F^−^ addition, beyond which further addition until 2 equiv. caused a constant decrease in intensity accompanied by a naked-eye color change from luminous yellow to bright green. The addition of 2 to 8 equiv.of F^−^ further caused an increase in emission, after which saturation is reached. From the changing nature of the emission curve, it was concluded that F^−^ caused deprotonation in the iridium complex. The two protons on the benzene ring of the complex are shifted upfield and after the addition of about 2 equiv. F^−^ caused the –NH signal to disappear. Strongly basic anions such as H_2_PO_4_^−^ and CH_3_COO^−^ caused deprotonation of highly acidity N-H protons of **127** and showed results that are similar to F^−^. Moderately basic anions such as Cl^−^, Br^−^, I^−^, and HSO_4_^−^ established remarkable H-bonding interactions without deprotonation taking place. The PL spectra were quite different from those of strongly basic anions and showed enhancement in the order Cl^−^ ˃ Br^−^ ˃ HSO_4_^−^ ˃ I^−^ indicating H-bonding interaction, although blue shifting in the emission spectra was common for all the above-mentioned anions.

Again, 3,5-dinitro benzoate(DNBA^−^) was able to quench the PL of complex **127** by 50% upon 1 equiv. of its addition, whereas upon 8 equiv. addition of the same reduced the PL intensity to 8% compared to the original complex. This “off-state” in situ formed 1:8 2-DNBZ complex served as an active sensor. The five different anions, namely F^−^, Cl^−^, Br^−^, I^−^, and HSO_4_^−^, were able to restore the quenched PL by replacing DNBA from **127** via a competitive binding and thus were able to switch on the luminescence of **127** (Figure 17b) [130]. The addition of almost 120 equiv. of each anion caused the increase in PL intensity by about 750%, 450%, 405%, 220%, and 118% for fluoride, chloride, hydrogen sulfate, bromide, and iodide, respectively, and the changes were observed via the naked eye (Figure 17c). From the emission bands of the fluoride titration profile shape, it was concluded that increasing concentration of F^-^ anion induced deprotonation in the iridium(III) complex (vide supra). Additionally, the association constants between **127**-DNBA and the anions were determined from the 1:1 binding model and were found to be: K_a_(Cl^−^) = 33 195, K_a_(HSO_4_^−^) = 17 804, K_a_(Br^−^) = 1572. Thus, it was concluded that based on the association, constant values **127**-DNBA discriminated between chloride and bromide.

There were a few more complexes of iridium(III) that could act as fluoride sensors. Huang et al. reported a dual emissive nanoprobe of iridium(III),**128** (Figure 17), which could form ultrasmall polymer dots in aqueous media and used this nanoprobe for selective, sensitive, accurate, and rapid detection of F^−^ in aqueous media as well as in biological systems [132]. In this regard, the tert-butyldiphenylsilyl group was attached to the iridium(III) complex, which could act as the F^−^ sensing signaling unit via quenching the phosphorescence. Nanoprobe **128** acted as a dual emissive ratiometric probe and accurately detected the F^−^ in live cells by determining the change in the ratio of F^−^-sensitive red phosphorescence from the iridium(III) complex to the F^−^ insensitive blue fluorescence from polyfluorene.

Again, Ma et al. reported a luminescent chemosensor, **129** (Figure 17), for tandem recognition of Al^3+^ and F^−^. Photophysical studies revealed the quenching of luminescing property upon the addition of F^−^ to **129** via hydrogen bonding interaction [133]. Again, luminesce is turned on in the presence of Al^3+^. So chemosensor **129** acted as an on-off-on sensor for F^−^ and Al^3+^.

Again, an interesting fluoride sensor was reported by Lee and co-workers. In this regard, they synthesized two cyclometalated Ir(III) complexes **130** and **131** (Figure 17), containing an o-carborane at the 4- and 5-position, respectively, in the phenyl ring of the pipyridine ligand [134]. X-ray crystal structure analysis of the closo-**130** revealed three C^N moiety chelates to Ir via fac-arrangement. Deboronation of the closo-carborane cage occurred upon the addition of F^−^ to both the non-emissive closo-**130** and closo-**131** complexes, which produced the corresponding emissive nido-carborane complexes (nido-**130** and nido-**131**). Therefore, these two carborane complexes could be used as turn-on emissive sensors for F^−^.

#### 2.2.2. Sensing of Cyanide

The detection of cyanide is an area of immense interest due to its extreme toxicity, even at very low concentrations. The limit of cyanide in water is 70 μg/L (in accordance with the World Health Organization (WHO)). So selective sensing and determination of cyanide is a contemporary area of research.

**Figure 18:**
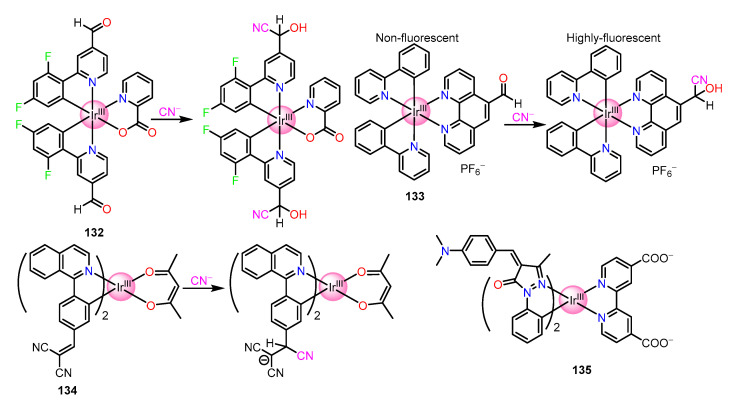
Structures of Ir(III) complexes **132**–**135**.

Reddy and coworkers reported a new cyanohydrin-forming phosphorescent iridium(III) complex **132** (Figure 18), which contains bis-2-(2,4- difluorophenyl)-4-formylpyridine ligands as the CN^−^ detecting unit [135]. Addition of 2 equiv. CN^−^ to acetonitrile solution of **132** caused a prominent orange-to-yellow color change accompanied by a drastic blue shift corresponding to 100 nm in the absorption maxima in the UV spectra of **132**. Other similar anions failed to cause a similar change, indicating a selective reaction of CN^−^ with the −CHO group. The association constant was estimated to be 1.029 × 10^5^ M^−1^ from the UV-titration experiments. The emission intensity of **132** was enhanced dramatically at 480 nm, with ~536-fold increment noted within 100 s, assisted with ~155 nm blue shift of the emission and a LOD value corresponding to 2.16 × 10^−8^ M. Furthermore, cyanohydrin formation was confirmed by ^1^H NMR, mass spectrometry, and FT-IR spectral studies which confirmed the vanishing of the aldehyde peak from the iridium complex and appearance of signals indicative of hydroxyl peak in the cyanide adduct. 

Yao group reported a cyclometallated phosphorescent complex of iridium, **133** (Figure 18), which was exploited for CN^−^ detection [136]. The complex was synthesized by cyanide alcoholized reaction on the C^N ligand (2-phenyl pyridine) and ancillary N^N ligand (1,10-phenanthroline-5-carboxaldehyde). The addition of CN^−^ caused cyanohydrin formation as evidenced by the vanishing of -CHO proton peat at δ 10.56 ppm and the appearance of a new peak at δ 8.60 ppm corresponding to the cyanohydrin proton signal. Further, the cyanohydrin formation was confirmed by FT-IR, mass spectra studies, UV−vis, and PL investigations. The PL spectra showed a turn-on response with 15-fold enhancement upon the addition of varying CN^−^ concentrations from 0 to 2 equiv. along with an alteration of the color of the solution (pale yellow to bright orange). The PL study revealed that **133** was selective towards CN^−^ over other reactive anions, and an excellent sensitivity corresponding to LOD of 1.23 μM in acetonitrile and H_2_O (95/5) was calculated. 

Hong and the group designed a specific molecular sensor **134** (Figure 18) with improved selectivity towards cyanide anion [137]. The complex offered a better reaction or binding site and could discriminate among various interfering anions. Through electrochemical manipulation, the two dicyanovinyl groups at the end of the phenylisoquinoline ligands became selective toward CN^−^. A 1:2 adduct is formed between dicyanovinyl and CN^−^ anion. The absorbance at 375 nm was found to decrease upon CN^−^ addition accompanied by naked eye color change. The strong electron-withdrawing dicyanovinyl group makes the photoluminescence negligible. The nucleophilic attack of CN^−^ at the β-position of the dicyanovinyl to form anionic species resulted in PL enhancement and characteristic blue shifting. Although the dicyanovinyl group is reactive to sulfides and thiols, the same is not reflected in PL as the enhancement in PL intensity in the presence of these two anions is only 15% of that from **134**-CN^−^ complex. ECL spectra of **134** are relatively weak. However, the addition of CN^−^ resulted in a strong ECL signal at 631 nm with 160-fold enhancement, whereas sulfides, thiols, and other anions failed to produce a similar pronounced response. The detection limit from the ECL experiment was estimated to be 0.04 μM. The ECL investigation technique was applied to tap water analysis which gave a rapid and successful detection of CN^−^.

A ratiometric upconversion luminescence (UCL) probe was developed by Li and co-workers for the selective detection of cyanide. In this regard, they synthesized NaYF_4_ (20%Yb, 1.6%Er, 0.4%Tm)-coated iridium(III) complex **135** (Figure 18), which inhibited the FRET method from UCL emission of the nanocrystals to the absorbance of the chromophoric iridium complex [138]. The limit of detection of CN^−^ by this nanocrystal probe was 0.18 μM towards CN^−^ and could be practically used for detection of CN^−^ in drinking water. This nanocrystal UCL probe is the first example of sensing and bioimaging of CN^−^ in living cells.

#### 2.2.3. Sensing of Phosphates

Phosphates are omnipresent in nature and are a fundamental part of our ecosystem. Its plays many vital roles in biological, environmental, and industrial processes. Due to the high hydration energies of phosphates, recognition and sensing was difficult task [139,140].

**Figure 19:**
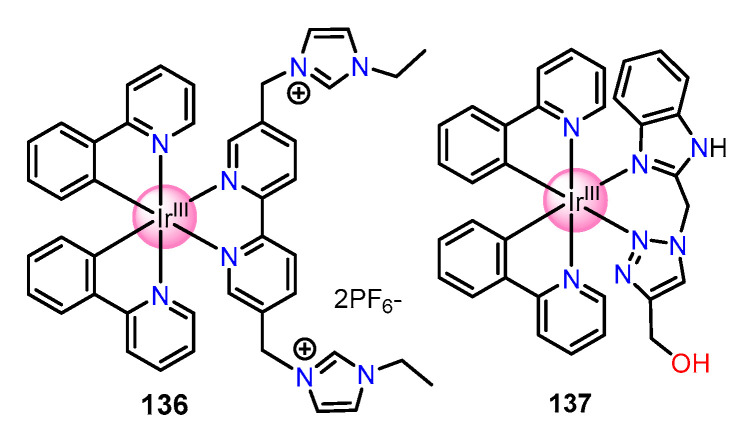
Structures of Ir(III) complexes **136**–**137**.

In 2022, our group synthesized a new bis-heteroleptic complex of iridium (III) **136** (Figure 19), which consisted of 1-ethyl-1H-imidazol-3-ium for the selective sensing of phosphates [141]. Complex **136** served as a lifetime-based sensor for phosphates in acetonitrile and was able to recognize and sense these two anions selectively. The lifetime of the free receptor complex **136** was 0.03543 μs which increased to 0.1323 μs and 0.2736 μs in the presence of HP_2_O_7_^3−^ and H_2_PO_4_^−^, respectively. PL intensities showed 13.7- and 8.5-fold increments for H_2_PO_4_^−^ and HP_2_O_7_^3−^ along with blue shifts in emission wavelength. Sensitive detection of these two anions was achieved with corresponding LOD values of 0.040 μM for H_2_PO_4_^−^ and 0.035 μM for HP_2_O_7_^3−^. From the NMR studies, it was established that the recognition occurred via CH···phosphate hydrogen bond (HB) interactions.

Again, PL-based detection of pyrophosphate via an iridium complex consisting of a new benzimidazole-substituted 1,2,3-triazole methanol ligand has been reported by Khatua and their group [142]. A three-fold increase in PL spectra of **137** (Figure 19) upon pyrophosphate titration was observed, and the subsequent binding constant of 8.6 × 10^7^ M^−1^ was calculated. Selective and sensitive detection of pyrophosphate was achieved in acetonitrile over competitive anions such as H_2_PO_4_^−^, ADP, ATP, and AMP. The LOD was assessed to be ~127 nM which indicated that complex **137** could detect pyrophosphate in the nanomolar concentration range without interference from other anions. Furthermore, the mechanistic investigations revealed that the acidic protons in the complexes formed H bonding with the solvent molecules and counter anions, while only the long-chain anions could interact with the complex. In addition, the PL increment was attributed to H-bonding interactions of the triazole C–H, methylene hydrogen, imidazole N-H, and hydroxyl groups with the pyrophosphate anion, as confirmed by ^1^H NMR. In addition, from the TDDFT calculation, it was established that in the **137**. Pyrophosphate adduct **137** becomes a better an efficient emitter as the energy gap between the ^3^MLCT–^3^MC is increased, and the emission occurs from the lower ^3^MLCT/^3^ILCT state. Furthermore, on performing cell imaging, it was found that the complex had low cytotoxicity.

#### 2.2.4. Sensing of Perchlorate

Perchlorate anions are water soluble and have an ionic radius and charge similar to iodide and so it could prevent the uptake and iodide amassing by the thyroid gland competitively. As a result, the deficiency of iodide in the body can be observed, which leads to impairment of the brain and goiter [143]. In addition, the relative stability of perchlorate anions is such that their availability is quite high in groundwater and surface water. So, the detection of ClO_4_^−^ in water is necessary because of its adverse effect on public health by taking food or drinking water [144].

**Figure 20:**
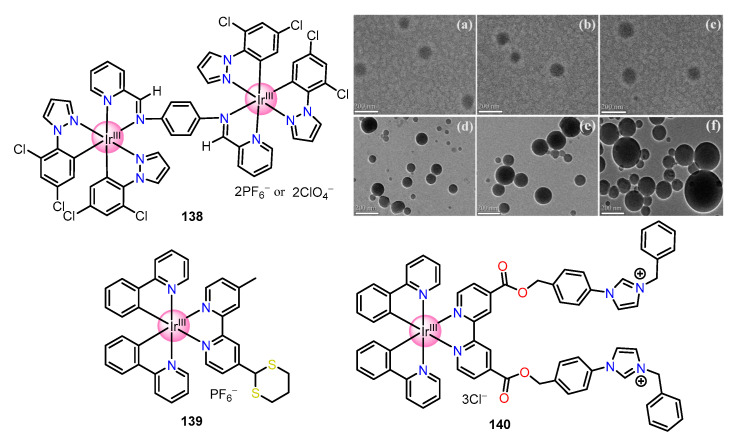
Structures of Ir(III) complexes **138**–**140** and TEM images of nanoparticles found from anion titration of (**a**) Free **138** (**b**) **138** + 10 equiv. HPO_4_^2−^; (**c**) **138** + 10 equiv. I^−^; (**d**) **138** + 3 equiv. ClO_4_^−^; (**e**) **138** + 5 equiv. ClO_4_^−^; (**f**) **138** + 10 equiv. ClO_4_^−^. [Adapted from reference [145]; Copyright © 2023, Royal Society of Chemistry].

A binuclear cationic iridium complex **138** (Figure 20) exhibited an efficient aggregation-induced emission in the presence of perchlorate anions [145]. The Schiff base complex displayed a fast, extremely selective, naked-eye, PL “turn on” response towards ClO_4_^−^ in both an aqueous medium and HeLa cells. The binuclear complex adds an advantage over mononuclear analogs by providing conformational flexibility. Interestingly, ClO_4_^−^-induced a phosphorescent turn-on in **138** with a decay lifetime of 0.24 μs, accompanied by a blueshift corresponding to 25 nm and 430 times increase in quantum yield. The other anions remarkably produced no substantial variation in the emission experiment performed in HEPES buffer. The phenomenon of the formation of ClO_4_^−^ induced nano aggregates was confirmed with the help of TEM and DLS experiments. TEM experiment reveals the average size was increased in the presence of ClO_4_^−^ while DLS showed an increase in the intensity of scattered light and particle size corresponding to 405 nm (Figure 20a–f). The LOD was calculated as 0.05 ppm from the intensity vs. concentration plot. Furthermore, confocal microscopy showed enhanced red phosphorescence in HeLa cells.

In 2016, Chao developed a strategy based on iridium(III) complex **139** (Figure 20) for imaging ClO_4_^−^ in the cell with strong red emission along with selective sensing of ClO_4_^−^ in an aqueous medium (1% DMSO) without the intervention of other ions [146]. The complex was weakly emissive at 595 nm. However, upon addition of ClO_4_^−^, the complex became emissive with a calculated LOD of 43.8 mM (via S/N = 3 methods), and hence it can be used as a luminescence “turn–on” probe for ClO_4_^−^ in HEPES buffer (10 mM, pH = 7.4). The UV absorbance titration spectra showed an increase in absorbance in the range of 400–550 nm, indicating a light scattering phenomenon caused due to nanoparticles formation in solution. The nanoparticle formation was further confirmed by DLS and SEM experiments which showed that the addition of 100 equiv. of ClO_4_^−^ induced the formation of larger aggregated nanoparticles having a particle size of 215 nm. It was further proposed that ClO_4_^−^ caused aggregation-induced emission enhancement (AIEE) of the weakly emissive iridium complex in water.

Further, Chao et al. modified and synthesized a water-soluble “turn-on” luminescence response iridium complex **140** (Figure 20) for ClO_4_^−^ via AIE [147]. Compared to probe **139,** which showed only 4-fold intensity enhancement with ClO_4_^−^, **140** exhibited a significant 250–fold enhancement at 632 nm. This unprecedented enhancement in emission beyond a threshold concentration of ClO_4_^−^ was credited to the electrostatic interaction between cationic **140** and anionic ClO_4_^−^. Additionally, 1:3 stoichiometry of binding was found, and the LOD was estimated to be 9.6 × 10^−7^ M. As observed for the complex **140**, the UV-Vis titration for **140** also showed an increase in absorption upon ClO_4_^−^ addition. Luminescence responses of **140** remained unperturbed to other anions, and a substantial red emission in the presence of only ClO_4_^−^ was observed. Further, macroscopic studies such as SEM, TEM, CLSM, and DLS were performed to obtain an understanding of the sensing mechanism, which unambiguously established the formation of induced aggregation nanoparticles due to ClO_4_^−^. Last of all, **140** was employed in cell–imaging of the complex in the presence of ClO_4_^−^ which displayed emission enhancement.

#### 2.2.5. Sensing of Chloride

**Figure 21:**
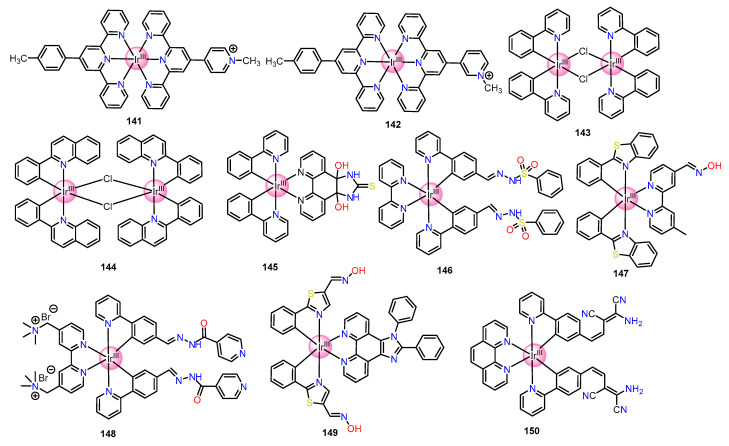
Structures of Ir(III) complexes **141**–**150**.

Williams et al. reported chloride induced luminesce quenching of isomeric bis-terpyridine functionalized iridium, complexes **141** and **142** (Figure 21) [148]. These two complexes differ based on the position of *N*-methylpyridyl substituents at the terpyridine units. Addition of aq. KCl led to a decrease in the PL intensities of both complexes, but the quenching was greater for complex **142** as compared to **141**. The lifetimes of emission were also perturbed for both complexes but to different extents. Thus, the two complexes acted as selective chloride sensors.

#### 2.2.6. Sensing of Nitrite

Two similar chloro-bridged iridium-based chemodosimeters **143** and **144** (Figure 21) for nitrite detection were reported by Schmittel and group [149]. The anion sensing occurs via the substitution of μ-dichloro diiridium complexes. The UV-Vis investigations of both the complexes were carried out in aqueous acetonitrile buffer, which showed absorption intensity enhancement in the presence of nitrite only, followed by an immediate color change for **143** (red to orangish yellow) and **144** (greenish to nearly colorless). The limit of detection from absorption was calculated to be 50 μM. The photoluminescence response of probes **143** and **144** to nitrite showed the opposite behavior. The addition of nitrite caused 350% enhancement in the PL spectra of **143** while **144** resulted in quenching of emission. Furthermore, the NMR and X-ray structure of NO_2_^−^ adduct with **143** and **144** revealed that the binding of NO_2_^−^ occurs via two different coordination modes (η^1^-nitrito-N and η^2^-nitrito-O, O’) and with distinct stoichiometries.

#### 2.2.7. Sensing of Carbonate/Bicarbonate

Pu et al. developed a novel luminescent “turn on” iridium (III) complex **145** (Figure 21) for HCO_3_^−^ and CO_3_^2−^ [150]. H-bonding interaction was operational between the hydroxyl group on **145** and the carbonate and bicarbonate anion, which caused the change in the PL spectra of the iridium(III) complex by altering the MLCT states of **145**. Only CO_3_^2−^ and HCO_3_^−^ were able to amplify the luminescence at 600 nm in THF at room temperature, while other anions failed to induce a similar response. The PL intensity was enhanced by 11 times and 14 times upon the addition of CO_3_^2−^ and HCO_3_^−^ respectively, with alteration of color (colorless to orang red) in both cases. A very low detection limit corresponding to 3.95 × 10^−8^ mol L^−1^ and 1.51 × 10^−7^ mol L^−1^ was calculated for CO_3_^2−^ and HCO_3_^−^. A 1:1 hosts-to-guest stoichiometry of binding was obtained from Job’s plot analysis and the mass spectrometry between **145** and the two anions separately. A quick response of <20 s was observed, and the association constants were calculated to be 2.67 × 10^−4^ and 2.27 × 10^−4^ for CO_3_^2−^ and HCO_3_^−^, respectively. Moreover, fluorescent test strips were also formulated for a cost-effective, simple operation and convenient detection of these anions. Overall, selective and sensitive detection of CO_3_^2−^ and HCO_3_^−^ was achieved for **145**.

#### 2.2.8. Sensing of Hypochlorite

Hypochlorite is recognized as a typical reactive oxygen species (ROS) and is crucial to various immune and pathological processes. It is widely utilized as a detergent, bleaching agent, disinfectant, and decontaminant. A conventional approach for designing ClO^−^ detecting probes includes a luminescent signaling unit (iridium), recognition site (ligand), and quencher (C=C, C=N), which controls the phosphorescent intensity of the probes to achieve the detection purpose. A series of “turn on” iridium-based receptors **146**–**150** (Figure 21) tailored with a phosphorescent quencher has been reported in the literature recently [151,152,153,154,155]. Fluorogenic compounds having unbridged C=N and C=C are either weakly fluorescent or non-fluorescent on account of excited state non-radiative decay caused by the isomerization of these bonds. Subsequently, upon reaction with ClO^−^ specifically, the C=N and C=C bonds in these complexes were oxidized to C=O and thereby generating the luminous nature of the probe.

## 3. Conclusions

The prime interest of this review is to give a comprehensive idea of some of the most exciting and pioneering research works of already developed phosphorescent ruthenium (II) and iridium (III)-based complexes and their applications as chemosensor for biologically/chemically/industrially important anions. These chemosensors are considered versatile materials for the detection of anions because they possess excellent photophysical properties such as large Stoke shifts, high quantum yield, longer lifetime, lesser auto-fluorescence, adjustable excitation and emission colors, commendable stability, higher biocompatibility. Here we have concisely amassed heteroleptic ruthenium (II) and iridium (III)-based complexes based on their recognition units in Table 1, as well as anion selectivity in different organic, semi-aqueous and aqueous solvents. 

Further, a substantial shortcoming in this direction is the decreased solubility of the designed organometallic receptor molecules in a 100% aqueous medium. A highly demanding research area of significant interest is the extraction of harmful anions generated as by-products from industrial wastes. However, fewer reports are documented in the literature for extraction or removal of inorganic anions from aqueous medium by ruthenium and iridium-based complexes as these anions have high hydration energy. Another drawback faced upon dealing with these complexes is the lower selectivity of iridium complexes which can be overcome in the future by proper modification of ligands. Therefore, this review is envisioned to provide extensive ideas and a significant amount of opportunity for researchers in the future to develop robust Ru(II)/Ir(III) complex-based chemosensors and their application for selective detection of anions in biological media, bioimaging, photo-activated therapeutic agents, and as a photo-activated catalyst.

## Figures and Tables

**Table 1:** Summary of different recognition/sensing unit-based sensors with accompanying references.

Recognition/Sensing Units	Authors and References
Triazole/iodo-triazole	Ghosh [52,53,54,55,56,57,58,59,60,61], Khatua [62,63], Anandan [64,65]
Imidazole/benzimidazole Amide/Sulphonamide/Picolinamide Pyrrol Urea Aldehyde phenol and catechol thiosemicarbazones dimesityboryl dicyanovinyl	Ye [69,70,71,72,74], Rau [73,131],Wang [79,80,81], Baitalik [82,83,84,85,86,87], Huang [127], Ghosh [141], Khatua [142], Chao [147] Rajagopal [90], Pinet [92], Sun [93], Lin [94], Zhong [96] Sessler [98,99], Anzenbacher [100] Das [103], Gunnlaugsson [104], Fabbrizzi [105], Yao [106] Schmittel [108], Yao [109,136], Reddy [135] Das [111] Chattopadhyay [112] Huang [116,121,125], Park [117], Huan [118,119,120], Lee [124,126] Hong [137]

## Data Availability

Not applicable.
